# Transcriptional Orchestration of the Global Cellular Response of a Model Pennate Diatom to Diel Light Cycling under Iron Limitation

**DOI:** 10.1371/journal.pgen.1006490

**Published:** 2016-12-14

**Authors:** Sarah R. Smith, Jeroen T. F. Gillard, Adam B. Kustka, John P. McCrow, Jonathan H. Badger, Hong Zheng, Ashley M. New, Chris L. Dupont, Toshihiro Obata, Alisdair R. Fernie, Andrew E. Allen

**Affiliations:** 1 Integrative Oceanography Division, Scripps Institution of Oceanography, UC San Diego, La Jolla, California, United States of America; 2 J. Craig Venter Institute, La Jolla, California, United States of America; 3 Department of Biology, CSU Bakersfield, Bakersfield, California, United States of America; 4 Department of Earth and Environmental Sciences, Rutgers University, Newark, New Jersey, United States of America; 5 Max-Planck-Institut für Molekulare Pflanzenphysiologie, Potsdam, Germany; MicroTrek Incorporated, UNITED STATES

## Abstract

Environmental fluctuations affect distribution, growth and abundance of diatoms in nature, with iron (Fe) availability playing a central role. Studies on the response of diatoms to low Fe have either utilized continuous (24 hr) illumination or sampled a single time of day, missing any temporal dynamics. We profiled the physiology, metabolite composition, and global transcripts of the pennate diatom *Phaeodactylum tricornutum* during steady-state growth at low, intermediate, and high levels of dissolved Fe over light:dark cycles, to better understand fundamental aspects of genetic control of physiological acclimation to growth under Fe-limitation. We greatly expand the catalog of genes involved in the low Fe response, highlighting the importance of intracellular trafficking in Fe-limited diatoms. *P*. *tricornutum* exhibited transcriptomic hallmarks of slowed growth leading to prolonged periods of cell division/silica deposition, which could impact biogeochemical carbon sequestration in Fe-limited regions. Light harvesting and ribosome biogenesis transcripts were generally reduced under low Fe while transcript levels for genes putatively involved in the acquisition and recycling of Fe were increased. We also noted shifts in expression towards increased synthesis and catabolism of branched chain amino acids in *P*. *tricornutum* grown at low Fe whereas expression of genes involved in central core metabolism were relatively unaffected, indicating that essential cellular function is protected. Beyond the response of *P*. *tricornutum* to low Fe, we observed major coordinated shifts in transcript control of primary and intermediate metabolism over light:dark cycles which contribute to a new view of the significance of distinctive diatom pathways, such as mitochondrial glycolysis and the ornithine-urea cycle. This study provides new insight into transcriptional modulation of diatom physiology and metabolism across light:dark cycles in response to Fe availability, providing mechanistic understanding for the ability of diatoms to remain metabolically poised to respond quickly to Fe input and revealing strategies underlying their ecological success.

## Author Summary

Oceanic diatoms live in constantly fluctuating environments to which they must adapt in order to survive. During sunlit hours, photosynthesis occurs allowing diatoms to store energy used at night to sustain energy demands. Cellular and molecular mechanisms for regulation of phytoplankton growth are important to understand because of their environmental roles at the base of food webs and in regulating carbon flux out of the atmosphere. In ocean ecosystems, the availability of iron (Fe) commonly limits phytoplankton growth and diatoms typically outcompete other phytoplankton when Fe is added, indicating they have adaptations allowing them to both survive at low Fe and rapidly respond to Fe additions. These adaptations may be unique depending on isolation from coastal or oceanic locations. To identify adaptive strategies, we characterized the response of a model diatom, *Phaeodactylum tricornutum*, to limiting Fe conditions over day:night cycles using a combination of gene expression analyses, metabolite, and physiology measurements. Major coordinated shifts in metabolism and growth were documented over diel cycles, with peak expression of low Fe expressed genes in the dark phase. Diatoms respond to limiting Fe by increasing Fe acquisition, while decreasing growth rate through slowed cell cycle progression, reduced energy acquisition, and subtle metabolic remodeling.

## Introduction

Organisms in earth’s photic zone experience diurnal cycles that influence physiology, metabolism, and growth in different ways. Photosynthetic organisms are particularly affected by light cycles since they depend on energy acquired from light harvesting to drive the biosynthesis of macromolecules essential for growth and metabolic demands in the dark. For these organisms, survival depends not only on sufficient illumination, but also on adequate supply of carbon dioxide and both macro and micronutrients. In marine ecosystems, availability of the micronutrient iron (Fe) plays a major role in controlling primary productivity, as Fe limitation is involved in regulating phytoplankton ecophysiology (at least seasonally) in 30% to 60% of sunlit ocean waters [1–4]. Large-scale Fe enrichment experiments typically result in diatom dominance of the phytoplankton biomass within days [5], implying that low density diatom stocks are maintained at low Fe concentrations [6], and indicating that certain diatoms have unique adaptive mechanisms to cope with low Fe poising them for rapid growth and ecological dominance upon Fe addition.

Fe is essential for the metabolism and growth of all organisms and diatoms respond in a variety of ways to Fe-limitation. For example, open-ocean species display lower concentrations of photosystem I, cytochrome b_6_f [7] and cellular pigments at the cost of light capture efficiency [8] and/or substitute Fe-containing enzymes with Fe-free equivalents, such as ferredoxin with flavodoxin or cytochrome b6 with plastocyanin [9–13]. Additionally, diatoms appear to induce reductive [14–16] and potentially non-reductive [17] high-affinity transport systems to acquire Fe. Some Fe-acquisition and homeostasis mechanisms have been shown to be dependent on the light:dark cycle in phytoplankton [18]. Fe stress also seems to impose a strain on overall proteome health that cells counteract by induction of proteolysis, chaperone expression and various reactive oxygen species (ROS) defense mechanisms [19–23]. The unique redox capabilities of Fe-containing metalloproteins are particularly essential in both photosynthetic and respiratory electron transport chains [24–26]. Therefore, decreases in Fe would be expected to impair cellular energetic processes during both light phases (photosynthesis) and dark phases (catabolism, respiration). Previous studies on the low Fe physiology of diatoms have been largely conducted under continuous illumination and/or sampled single time points and it is currently unknown how Fe-limitation affects cellular processes including cell division and metabolic shifts over diel cycles.

High resolution sampling has revealed the extent to which physiological states vary over diel cycles and how these state changes are accompanied and regulated by transcriptome shifts in phytoplankton [27–30]. Here, we provide the first global cellular insights into metabolic shifts between diel cycles and at varying Fe concentrations in the model pennate diatom *Phaeodactylum tricornutum* using a time course sampling the physiology, metabolome, and transcriptome at high resolution. We investigate the effect of steady-state Fe-limitation on the temporal expression of genes involved in diverse cellular processes including Fe acquisition, endocytosis, cell cycle progression and division, ribosome biogenesis, light harvesting complex assembly, and primary carbon and nitrogen metabolism. We greatly expand the catalog of highly Fe sensitive genes putatively involved in novel Fe acquisition mechanisms [19], and identify specific transcription factors and gene regulatory mechanisms utilized by the model diatom *P*. *tricornutum* to adapt to Fe-limitation. Though *P*. *tricornutum* is not ecologically dominant, it is a useful model for Fe studies in diatoms because it has a low susceptibility to Fe limitation, grows well at ecologically relevant Fe concentrations, and has the highly iron responsive gene set possessed by oceanic but not coastal diatoms [14,20]. Taken together, these data provide an in-depth view of the global cellular processes operating over the diel cycle, leading to physiological acclimation to low Fe conditions.

## Results and Discussion

### Fe-limited response of *P*. *tricornutum*

**Fig 1:**
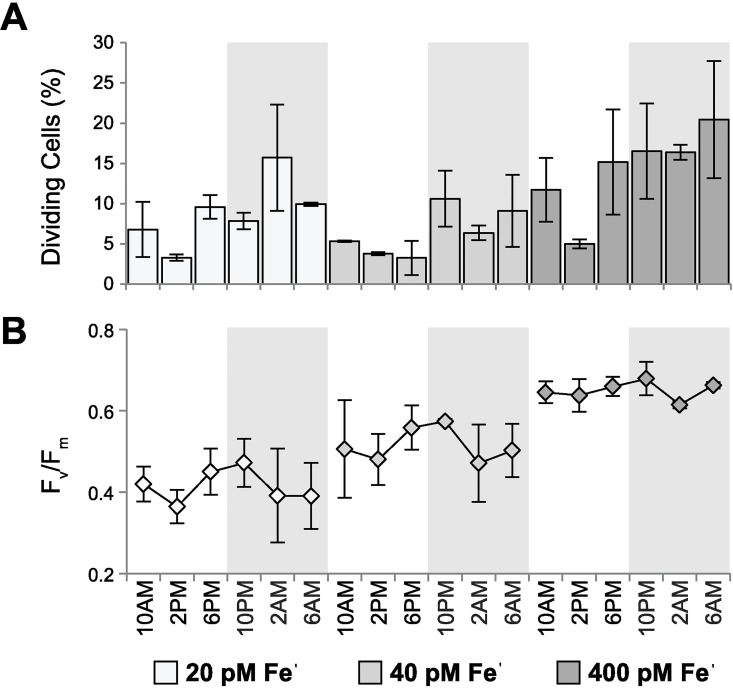
Physiological response of *P*. *tricornutum* to low Fe across diel cycles. Diel patterns in cell division and fluctuations of F_v_/F_m_ in *P*. *tricornutum* grown at 20 pM, 40 pM, and 400 pM Fe′ over 12:12 L:D cycles, lights on = 9am, lights off = 9pm. (**A**) Bar plot of the time point averaged percentage of dividing cells shown with standard deviation across biological replicates. (**B**) Time point averaged F_v_/F_m_. Legend is shared for both plots and shaded background indicates dark time points.

Steady-state cultures of *P*. *tricornutum* were grown at three different iron (Fe) concentrations, two of which were limiting at 20 pM and 40 pM Fe′ (low Fe) in biological duplicate, and non-limiting Fe conditions at 400 pM Fe′ (high Fe) in biological triplicate over a 12:12 light:dark cycle. Cultures were sampled over a complete diel cycle, every 4 hr for 28 hr (7 time points) resulting in a total of 49 time points sampled (S1 Table). Relative to high Fe cultures, Fe limitation reduced growth rates by 25 and 28% at 40 pM Fe′ and 20 pM Fe′ respectively (Table 1, S1–S7 Figs). At all Fe conditions, cell division peaked during the dark phase (2–6 AM; Fig 1A). Generally, cell division in phytoplankton is entrained to light:dark cycles, however several diatom species including *P*. *tricornutum*, have been difficult to synchronize based on light:dark cycles alone [30–36]. These observations are consistent with the relatively small degree of growth synchronization observed in our cultures where percentage of dividing cells reached a maximum of c. 20% (Fig 1A).

**Table 1:** Physiological response of *P*. *tricornutum* to varying Fe and illumination. Average growth rates (biological duplicate for 20 pM and 40 pM Fe′, biological triplicate for 400 pM Fe′ with standard deviation), average divisions per day, and F_v_/F_m_.

	Low Fe		High Fe
	20 pM Fe′	40 pM Fe′	400 pM Fe′
**Specific growth rate (μ)**	0.57 (± 0.14)	0.81 (±0.02)	1.07 (±0.08)
**Divisions per day**	0.82	1.16	1.54
**Average F**_**v**_**/F**_**m**_ **(**± SD)	0.412 (± 0.070)	0.512 (± 0.075)	0.642 (± 0.011)

In addition to reduction in growth rates, Fe availability impacted F_v_/F_m_, (the maximum potential quantum efficiency of PSII). Average F_v_/F_m_ of *P*. *tricornutum* grown at 400 pM Fe′ was near the theoretical maximum (F_v_/F_m_ = 0.642), indicating high photosynthetic efficiencies, which decreased on average by 20% and 36% (in 20 and 40 pM Fe′ respectively; Table 1).

Here, reductions in F_v_/F_m_ result from both the reduced capacity to synthesize Fe rich components of the photosynthetic electron transport chain (PSII, cytochrome *b*_*6*_*f*, cytochrome oxidase, PSI and ferredoxin), and the production of photoprotective chlorophyll-protein complexes that are energetically disconnected from the photosynthetic electron transport chain particularly under non-limiting macronutrient concentrations [4, 37–39]. Diel fluctuations in F_v_/F_m_ were consistent in all Fe conditions with a mid-day minimum (likely due to photoinhibition), maxima at 10PM (2 h after transition to darkness), followed by a significant decline by 2AM (Fig 1B). Nocturnal decreases in F_v_/F_m_ are caused by chlororespiration mediated reduction in the plastoquinone (PQ) pool and resulting back-transfer of electrons which decreases the overall efficiency of electron transport at PSII [38], corroborated by significant rates of chlororespiration previously measured in *P*. *tricornutum* [40–43]. There was a modest recovery of F_v_/F_m_ before dawn across Fe concentrations, which occurs as cells deplete the pool of respiratory substrates [38]. Together, growth rate measurements and physiological fluorescence confirm that *P*. *tricornutum* grown at 20–40 pM Fe′ experience Fe limitation, and are energetically impaired relative to the Fe replete (400 pM Fe′) cultures. These physiological patterns are further explored in the context of diel patterns in metabolite and transcript profiles.

### Global transcriptome dynamics and diel expression of highly Fe responsive genes

**Fig 2:**
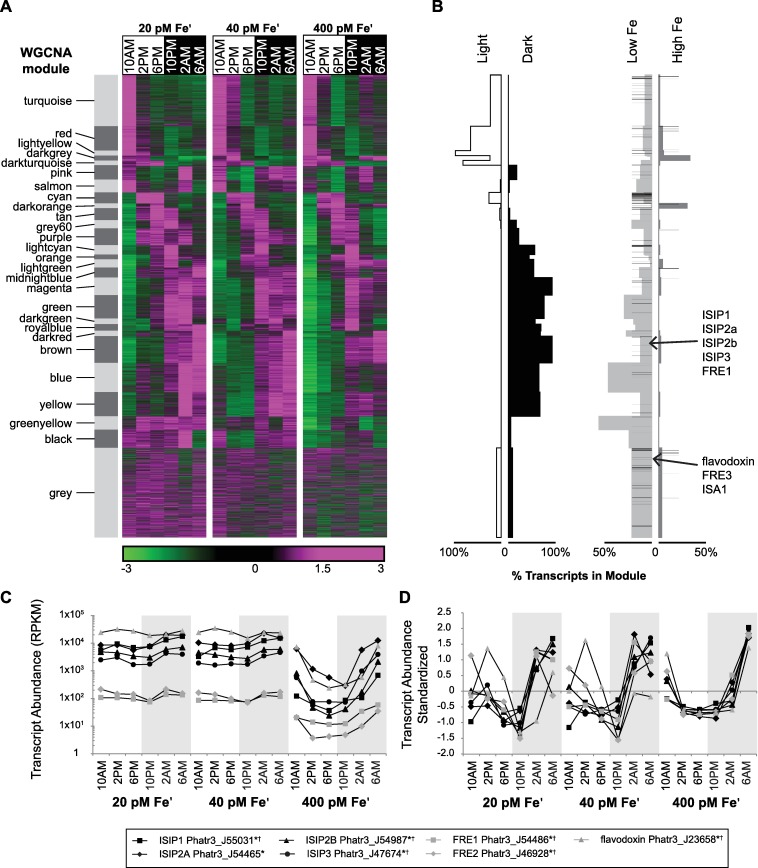
Genome wide transcriptome dynamics and transcript level expression of Fe status biomarker genes. The transcriptome of semi-continuous cultures of *P*. *tricornutum* grown over diel cycles (12:12) at Fe limited (20 and 40 pM Fe′) and Fe replete (400 pM Fe′) conditions is shown. (**A**) Heat map shows standardized transcript abundance (standardized RPKM across Fe conditions) for each gene. Genes organized into co-expression modules (n = 8418 genes, labeled with module color names) and ordered by hierarchical clustering results. (**B**) Percentage of genes from each expression module belonging to response type groups (light, dark, low Fe, or high Fe determined with Skillings-Mack) indicated. Black bars indicate genes previously identified as Fe-responsive in [19]. Identities of genes and module and response type assignments can be found in S1 Dataset. (**C**) Time point averaged transcript abundance (RPKM) is shown, plotted on a log scale, error bars omitted for clarity (**D**) Standardized transcript abundance (calculated independently for each Fe condition) is also plotted to illustrate dark phase expression maxima. Legend includes gene names and Phatr3 PIDs. Background shading indicates dark time points. *Binned low Fe by Skillings-Mack, †Binned low Fe by EdgeR

The transcriptome was analyzed using two principal methods, expression clustering and differential expression (DE) binning (Methods). First, we explored genome wide co-expression by using Weighted Gene Co-Expression Network Analysis of RPKMs (WGCNA; [44–46]). WGCNA yielded 27 co-expression clusters or “modules” from a filtered set of the active transcriptome (n = 8499) and containing only nuclear-encoded genes, with modules ranging in size from 81 to 937 genes (Fig 2, S1 Dataset). For DE binning, genes were determined to be expressed as a function of Fe′ or illumination status by assignment to 8 different response types, characterized by elevated transcript abundance under those conditions. These response types were “low Fe” (20 pM and 40 pM Fe′), “high Fe” (400 pM Fe′), “light” (10 AM, 2 PM, 6 PM), and “dark" (10 PM, 2 AM, 6 AM) with the four remaining bins being genes assigned to both an Fe or light condition (“dark, low Fe”, “dark, high Fe”, “light, low Fe”, “dark, low Fe”). The two DE approaches utilized (non-parametric Skillings-Mack (SM) test on reads per kilobase of transcript per million (RPKM) values and the LR test in EdgeR, see methods) returned different total numbers of genes, but several genes (n = 2,389) were identically binned by both tests yielding response type calls we considered to be high confidence (Table 2, S1 Dataset). The main difference in the gene sets returned between the different tests were that the SM test returned more significantly DE genes in the dark and at low Fe, and the LR test returned more significantly DE genes in the light and at high Fe, differences which are likely attributed to variation in the normalization procedures (methods). The percentage of each module assigned to response types by the Skillings-Mack test are shown (Fig 2A and 2B).

**Table 2:** Differentially expressed gene totals. Total number of genes determined to be significantly differentially expressed (p < 0.05) by the Skillings-Mack test and the LR test in EdgeR (EdgeR) and by both tests. Total number of genes determined to be differentially expressed only by one method or the other (Total Unique) are also shown. The three most abundant unique response types for each test are shown in bold. Ratios of Low Fe and High Fe genes expressed in the dark relative to light are calculated for each test.

	Total Significant			Total Unique	
Response Type	Both (High Confidence)	SM	EdgeR	SM	EdgeR
**Dark only**	1155	2616	1390	**1461**	235
**Light only**	722	897	1549	175	**827**
**Low Fe only**	250	1240	322	**990**	72
**High Fe only**	100	147	440	47	**340**
**Dark, Low Fe**	122	732	135	**610**	13
**Dark, High Fe**	3	27	31	24	28
**Light, Low Fe**	18	99	43	81	25
**Light, High Fe**	19	24	299	5	**280**
***Total*:**	*2389*	*5782*	*4209*	*3061*	*1447*
**(# Dark, Low Fe)**	6.7x	7.4x	3.1x		
**(# Light, Low Fe)**					
**(# Dark, High Fe)**	0.2x	1.1x	0.1x		
**(# Light, High Fe)**					

Though there were differences in total number and response type distribution of DE genes returned with both statistical approaches, there were consistently more “dark, low Fe” genes relative to “light, low Fe” genes: a pattern that did not hold at high Fe (Table 2). This points to the dark as a particularly important time for transcriptional output in cells grown at low Fe. Genes which had elevated dark phase gene expression at low Fe are likely to be involved in nocturnal metabolic and cellular processes required for growth in these conditions. Known Fe responsive genes such as Fe starvation inducible proteins (ISIPs), flavodoxin, ferric reductases (FRE) were expressed strongly at low Fe and also during the dark phase at all Fe conditions suggesting the promoters of these genes are sensitive to both Fe status and illumination phase (Fig 2C and 2D). There were Fe-specific differences in the timing of nocturnal induction. At low and med Fe, elevated transcript levels were detected at 2AM, preceding maximum induction of the same genes at 6AM in Fe-replete cultures, possibly as a response to reduced cellular Fe quotas at Fe-limitation (Fig 2D).

The significance of nighttime induction of Fe-responsive genes is unknown. Previously it has been shown that ISIP2a has a role in Fe uptake at the cell surface in *P*. *tricornutum*, and ISIP2a protein levels and Fe-uptake rates are elevated at night [17]. This, combined with diel regulation of several Fe-responsive genes points to the dark phase as an important time for Fe metabolism. Diel patterns in Fe metabolism have been observed in other marine phototrophs. For example, the marine diazotroph *Crocosphaera watsonii* recycles intracellular Fe reserves for synthesis of day or night specific metalloenzymes (“hotbunking”) as a strategy to reduce its overall Fe requirement, [47]. Also, *Ostreococcus tauri* expresses ferritin nocturnally in order to store intracellular Fe recovered from damaged/oxidized iron-binding proteins [18]. Ferritin is used for iron storage in some bloom-forming diatoms [48] and may be similarly nocturnally expressed in these species, though no diel pattern was detected for ferritin in our experiments with *P*. *tricornutum*. However, nocturnal expression of other Fe-sensitive genes (like ISIPs) in *P*. *tricornutum* may occur in order to scavenge Fe from damaged proteins (like *O*. *tauri)*, and/or to reuse cellular Fe during diel protein turnover (as in *C*. *watsonii*), even under Fe-replete conditions.

In order to identify other candidate genes involved in specific mechanisms to acquire and maintain cellular Fe and gain additional insight into the significance of nocturnal expression of Fe-sensitive genes, the complete low Fe response type gene set (significant by either SM test or EdgeR, n = 2090) was extracted, clustered hierarchically, and surveyed for gene functions (S2 Dataset, S8 Fig). Improved detection of low Fe sensitive transcripts in this experiment over previous efforts can be attributed to both improvements in transcriptome sequencing coverage, and increased resolution of sampling frequency [19]. This gene set contained several annotated genes not previously known to be Fe sensitive, with putative roles in Fe sequestration and transport, including a highly expressed ZIP Zinc transporter (ZIP1; Phatr3_J52343), and two genes that encode heavy metal-transporting P-type ATPases (Phatr3_EG02342, Phatr3_Jdraft391). One of the most highly induced low Fe genes encoded a protein with a hemerythrin-like domain and a CHY zinc finger domain at the C-terminus (Phatr3_J12097). Hemerythrin domains are found in Fe-binding regulator proteins in both animals and plants, raising the intriguing possibility that this gene has a similar role in *P*. *tricornutum* [49–50]. Phytochelatin synthase (PCS; Phatr3_J48175) was among the genes highly co-expressed with the ISIPs. Phytochelatins are peptides that play an important role in metal detoxification in plants and fungi and are synthesized by PCS from glutathione and glycine [51]. Nighttime induction of this enzyme in Fe-limited *P*. *tricornutum* could aid in contributing to defense against oxidative stress; directly or through sequestration, storage, and intracellular buffering of Fe, reducing free radical generation from temporal fluctuations in excess free Fe [52]. This would be particularly important for cells adapted to Fe-limitation, which are already heavily impacted by oxidative stress from impaired photosynthetic and mitochondrial electron transport [4].

Other genes found within the highly Fe-responsive set and strongly induced at the transcript level at night and at low Fe were two cathepsins (Phatr3_J25433, Phatr3_J4936) and a v-SNARE protein (Phatr3_J40521/Phatr3_EG02577). Cathepsins are proteases that are active in the low pH of lysosomes, while v-SNARE proteins are involved in intracellular vesicle transport. Vesicle trafficking is a major cellular activity that occurs during normal cellular function to transport proteins between organelles, but it is particularly important during endocytosis, exocytosis, and autophagy, when molecules are transported to the lysosome for degradation. Genes with specific roles in these processes are well-studied in other organisms and homologs of many of these genes were identified in the *P*. *tricornutum* genome. Homologs of genes involved in endocytosis, exocytosis, and autophagy including genes of the exocyst complex, cytosokeleton components, and additional SNARE proteins, were upregulated at low Fe during the dark phase [53] (S3 Dataset). Other transcripts for genes with endocytosis and vesicular sorting functions, such as endosomal coat protein subunits, adaptins, and putative GTPases from the dynamin and Rab family, and members of the ESCRT complex were upregulated in a similar fashion, as were transcripts with established homology to genes with known roles in autophagy. Finally, genes with roles in glycosylation, oligosaccharide processing, and phosphoinositide metabolism, all important in membrane trafficking and sorting, were also upregulated at low Fe (S3 Dataset). Overall, this illustrates that the processes of endocytosis, exocytosis, and autophagy and associated intracellular transport of myriad cellular components occurs mostly during the dark phase in *P*. *tricornutum*, reflecting at least in part a higher degree of lysosomal protein degradation. ƒ trafficking and adaptive significance of these processes for diatoms grown under these conditions. Lommer et al. [20] hypothesized that endocytosis has a role in Fe acquisition, based on the identification of a conserved endocytosis motif in ISIP1 from *Thalassiosia oceanica*. Our results provide additional support for this hypothesis and point to this cellular process as an evolutionarily conserved strategy among diatoms to regulate cellular Fe. Further, our data identify candidate genes specifically involved in that process, such as the cathepsins and v-SNAREs.

There were several transcription factors (TF) identified among the low Fe responsive gene set [54](S2 Dataset). Of the three most highly induced TFs, one was from the C2H2 Zn finger family (CCHH11; Phatr3_J38018), one was from the Myb family (Myb1R_SHAQKYF3; Phatr3_J44256), and another was a sigma factor (sigma70.1a; Phatr3_J14599) predicted to be targeted to the chloroplast, presumably to regulate the chloroplast genome (S2 Fig, Dataset). Several other TFs were also induced at low Fe but to a lesser extent (S9 Fig). We hypothesize at least the most highly upregulated TFs could be involved in regulating downstream cellular responses to low Fe. CCHH11 was the most strongly upregulated gene at low Fe with an average of 453 reads per kilobase of transcript per million mapped (RPKM), 171 RPKM, and 10 RPKM detected at 20 pM, 40 pM, and 400 pM Fe′ respectively (S2 Fig), but lacked clear diel expression patterns. A transmembrane domain was detected at the C-terminus of CCHH11 that did not overlap with the predicted DNA binding domain (S2 Fig). Often, TFs which possess a transmembrane domain are cleaved proteolytically to become liberated and active in regulating gene transcription in a process known as regulated intramembrane proteolysis (Rip; [55]). Dramatic upregulation of transmembrane-containing CCHH11 at low Fe, combined with our observed elevated expression of endocytotic and lysosomal genes at low Fe, could implicate Rip as a previously unknown mechanism for *P*. *tricornutum* to respond and cope with low Fe.

This dataset expands the catalog of putative Fe acquisition and Fe regulatory gene candidates, but the cellular response to Fe-limited growth is not limited to Fe acquisition and homeostasis. The more global cellular response to low Fe, evidenced by the large number and diverse patterns of genes assigned to the low Fe response type, is also important to investigate low Fe adaptation at the genome scale (S2 Dataset).

### Transcriptomic hallmarks of reduced growth at low Fe and successional expression of cell cycle regulators

**Fig 3:**
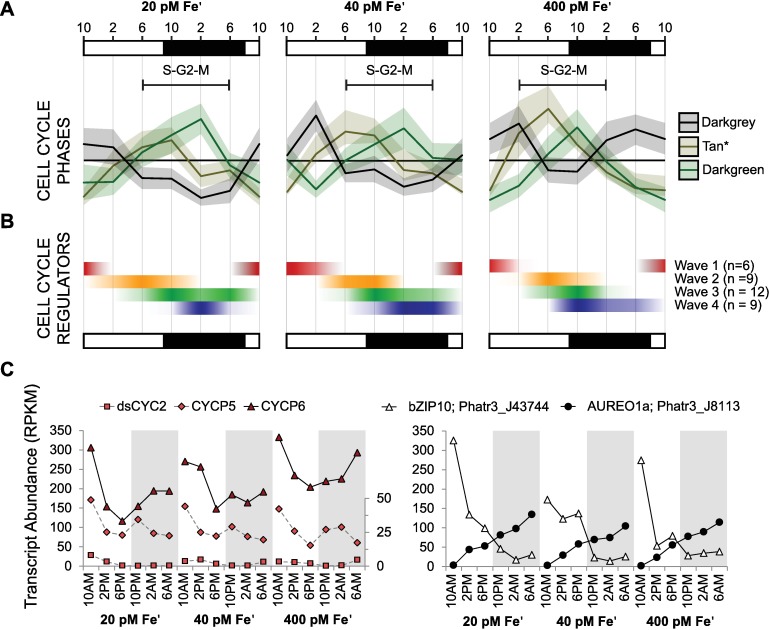
Expression of cell cycle progression-related genes, ribosome biogenesis, and cyclins and cyclin-dependent kinases (CDKs). (**A**) Data show average standardized transcript abundance (across Fe conditions) in WGCNA co-expression modules enriched for gene functions associated with cell cycle progression (tan–selected genes see S1 Dataset, darkgreen) and ribosome biogenesis (darkgrey) genes at 20, 40, and 400 pM Fe′ with shaded area indicating one standard deviation (S1 Dataset for gene IDs). (**B**) Cyclin and CDK transcript abundance. Waves of co-expressed cyclins and CDKs were identified with hierarchical clustering (S10 Fig). Standardized cluster averages (within each Fe condition, positive values only) are shown. (**C**) Transcript abundance (RPKM) of wave 1 cyclins and dsCYC2-regulating transcription factors (bZIP10 and Aureo1a) are shown. Dashed lines indicate data are plotted on the secondary y-axis.

To characterize Fe and diel-modulated differences in expression, biological roles of genes within co-expression modules and response types were surveyed by calculating over and under-representation of Gene Ontology (GO) annotations (S2 Table). Several modules were disproportionately enriched in genes attributed to various cellular processes, such as growth and division (S2 Table). Core histone transcripts (H2A/B, H2, H4), main nucleosome components, were strikingly abundant in the tan module (S2 Table, S1 Dataset). In *Drosophila*, expression of histone mRNAs is tightly constrained to a brief pulse during S-phase [56], and a similar correlation has been observed in the diatom *Thalassiosira pseudonana* [57]. Manual inspection of annotated genes in the tan module revealed that several genes were functionally associated with S-phase, indicating that peak timing of expression in this module may correspond to a time in the diel cycle when the majority of the *P*. *tricornutum* population is progressing through S-phase (S1 Dataset). Key transcripts of mitosis-related genes were found in the darkgreen module (S1 Dataset), including the cell division cycle protein 20 (CDC20, Phatr3_J12783): a highly conserved key activator of the anaphase promoting complex specifically expressed during the G2-to-M-phase in *P*. *tricornutum* [58]. Peak expression of the darkgreen module lagged shortly behind S-phase expression (selected tan module genes, S1 Dataset), as would be expected as the population progresses through the cell cycle (Fig 3). Predominantly, these S-phase and M-phase genes were expressed during the light to dark transition correlating with the timeframe when cells were dividing (Fig 1B and Fig 3). Fe-replete cultures expressed cell cycle phase-specific genes earlier and over a shorter period of time (2PM, 12hr) than what was detected in Fe-limited cultures (6PM, 16hr) (Fig 3A). This indicates that at high Fe, the population is both able to begin dividing sooner and is more highly synchronized than cells grown at low Fe. We hypothesize that this delay in timing of cell cycle progression in low Fe cultures, inferred from mRNA levels of phase marker genes, is a consequence of reduced energetic status in Fe-impoverished and photosynthetically compromised cells.

Cell growth (leading to division) depends on protein synthesis by ribosomes, and reductions in ribosome assembly and translation are often among the first and most drastic responses when cells experience environmental stress [59,60]. In our experiments, two modules (turquoise, darkgrey) with similar overall patterns of expression were enriched in genes required for ribosome biogenesis (S2 Table). The darkgrey module was nearly completely comprised of transcripts for almost all of the ribosomal proteins (RPs) encoded in the *P*. *tricornutum* genome (S1 Dataset). Peak expression of RPs occurred largely during the illuminated phase of the diel cycle, and in an inverse pattern to expression of cell cycle progression genes, illustrating that the cell segregates expression of genes essential for growth and division temporally (Fig 3). RPs are some of the most highly expressed mRNAs in the *P*. *tricornutum* genome, and though they are highly expressed overall, they were less highly expressed at low Fe (S1 Dataset). Reduction of ribosome biogenesis, and subsequent decreased protein biosynthesis could explain reduced growth rates at low Fe.

Progression through the cell cycle is regulated by cyclins and cyclin-dependent kinases (CDKs). Cyclins and CDKs are evolutionarily conserved in eukaryotes, and cyclins have expanded in diatoms, presumably to regulate cell cycle progression in response to varied environmental signals [61–62]. Over diel cycles, four distinct waves in expression of cyclins and CDKs were identified with expression of one wave of these cell cycle regulators limited to the day (wave 1) while most other cyclins and CDKs (waves 2, 3, 4) were more highly expressed during the light-dark transition period. Cyclin/CDK wave 1 contained the G1 marker cyclin CYCP6 and the diatom specific cyclin dsCYC2 known to be involved in regulating the light-dependent onset of G1 phase in *P*. *tricornutum*. This finding, combined with the observation of decreased proportion of dividing cells detected during the light indicate that the majority of the population is in the G1 phase at this time. Dark phase expression of the other waves of cyclins/CDKs is consistent with observations of increased cell division and the conclusion that this time period is particularly active for cell cycle progression (Fig 3B, S10 Fig). Like the S- and M-phase modules, the window of peak cyclin/CDK expression (waves 2–4) was shorter in Fe-replete cultures than in Fe-limited cultures, corroborating a higher degree of synchrony in Fe-replete populations. The timing of cyclin/CDK waves 2 and 3 correlated with the S-phase and M-phase enriched modules respectively, leading us to investigate whether these cyclin/CDK waves were cell cycle stage or transition specific. Indeed, cyclin/CDK wave 2 contained several regulators previously shown to be expressed at the G1-S boundary in *P*. *tricornutum* such as CYCL, dsCYC6, hCDK2, and hCDK5 and cyclin/CDK wave 3 contained marker genes for the G2-M transition such as CDKA2 and CYCB1 [62–63]. However, each wave also contained genes previously assigned to the opposite phase transition (S10 Fig, [62]). In the previous study, Huysman et al. [62] obtained a much higher degree of synchrony with prolonged dark exposure, so the observed discrepancy is most likely attributed to heterogeneity in our mildly synchronized populations. However, the temporal segregation in G1-S and G2-M cyclin expression we observed suggests their transcript level expression dynamics are more nuanced than has been demonstrated previously, illustrating our limited understanding of cell cycle regulation, and underscoring the need for an increased number of observations and experiments to continue to elucidate cyclin/CDK specificity and roles in diatom cell cycle.

Diatom cyclin and CDK expression is affected by nutrient supply, with previous work indicating that dsCYC10 is sensitive to phosphate availability [62]. In this study, dsCYC10 (Phatr3_50251) was also up-regulated at low Fe (with high confidence), supporting the idea that it may sense or relay the status of multiple nutrients during the cell cycle [62]. Other cell cycle regulators were also sensitive to low Fe, however few dramatic Fe-specific modulations in transcript abundance were detected (S10 Fig). At low Fe, these genes may be modestly upregulated in order to drive cell cycle progression forward in particularly energetically impaired cells.

Transitions in illumination state are strong signals that are associated with transcriptome state shifts in diatoms [28]. In *P*. *tricornutum*, light is a driver of cell cycle progression through the expression of light sensitive cyclins such as dsCYC2 (cyclin/CDK wave 1), regulated transcriptionally by AUREOCHROME1a (AUREO1a, Phatr3_J8113) and bZIP10 (Phatr3_J43744) transcription factors [64]. Strong temporal dynamics in the transcript abundance of AUREO1a and bZIP10 were observed under diel cycles (Fig 3B). Transcripts of AUREO1a steadily accumulate throughout the diel cycle, reaching maximum abundance prior to the onset of illumination indicating that the dark-light transition represses transcription of AUREO1a. In contrast, bZIP10 is at maximum transcript abundance directly following the dark-light transition, and is minimally expressed during the dark. Opposing diel expression patterns by the two TFs that regulate dsCYC2 production, could indicate a functionally distinct role to coordinate cell division with both the light-dark and dark-light transitions to ensure cell cycle progression is synchronized fully to light cycles, and not just the onset of illumination. Despite the detectible diel cycling of dsCYC2, and the previously described role of this gene in cell cycling, we noted that overall mRNA levels of this gene were extremely low (<5 RPKM) relative to other similarly expressed cyclins (Fig 3B). This suggests that cyclins other than dsCYC2, such as CYCP5 and CYCP6, which are drastically modulated in our experiments, also have important roles in linking cell cycle progression to the dark-light transition. Though *P*. *tricornutum* does not synchronize tightly to diel cycles, there is a subtle natural synchrony evidenced by the dark phase increase in proportion of dividing cells, and peaks in expression of the majority of cell cycle regulators (including cyclins and CDKs). The degree of synchronization is dependent of the Fe status of the population, with rapidly growing (Fe-replete) populations not only dividing earlier, but over a shorter period of time in the diel cycle than Fe-limited populations. Overall, these results provide additional details and mechanistic insight into how nutrients and light drive populations to become synchronous with the light cycle [65–66].

It is possible that there are biogeochemical consequences to this slowed growth at low Fe. Generally, slower growth results in a reduced rate of progression through the cell cycle, and as a consequence cells spend a longer period of time in M-phase. This is biogeochemically and ecologically significant, since M phase is the primary period of silica deposition [67]. Some diatoms grown in Fe-limiting conditions with sufficient silicic acid availability (e.g., HNLC regions) have more heavily silicified cell walls and are therefore more efficient at long term carbon sequestration from the surface ocean [12, 68–72]. Although *P*. *tricornutum* is a lightly silicified diatom, the Fe-specific and diel regulation of these (likely cell cycle driven) processes are likely to be similar within more heavily silicified diatoms.

### Regulation of the light harvesting complex and partial downregulation at low Fe

Up to 80% of the cellular Fe requirement is associated with photosynthesis in photoautotrophic cells, where Fe is bound to various cytochromes, and Fe-S cluster proteins of the photosynthetic electron transport chain [26]. Fe depletion in phototrophs impairs photosynthetic efficiencies, leading to increased production of damaging reactive oxygen species (ROS) when photosynthetic light harvesting exceeds the capacity of photosynthetic electron transport [73]. Reorganization of the light harvesting machinery is expected be an important aspect of acclimation to shifting Fe and light conditions. To better define how this is accomplished, the temporal expression dynamics of key light harvesting genes were investigated.

Transcripts encoding for both pigment biosynthesis and light harvesting complex (LHC) proteins were found nearly exclusively in the similarly expressed cyan (n = 199) and darkorange (n = 81) modules, which peak in the late afternoon (2PM–6PM; Fig 2). The cyan module contained the complete pathway for porphyrin and chlorophyll metabolism, and several steps in terpenoid backbone biosynthesis, including genes such as polyprenyl synthetase (Phatr3_J19000), geranylgeranyl reductase (Phatr3_J31683), and chlorophyll synthase (Phatr3_J12807). Together, the cyan and darkorange modules contained nearly all annotated antenna proteins of the LHC superfamily which bind pigment and transfer absorbed light energy to the reaction center complexes. These modules also contained transcripts for nuclear-encoded components of photosystem II (PSII) such as PsbM (Phatr3_J55057), PsbO (Phatr3_J20331), and PetJ (Phatr3_J44056). A large proportion of the darkorange module (LHC transcripts), was downregulated at low Fe, consistent with findings from previous transcriptome studies of Fe-limited phytoplankton [19–20]. In contrast, several genes contained within the cyan module (pigment biosynthesis genes) were upregulated in Fe limiting conditions. The overall downregulation of the LHCs and upregulation of pigment (and specifically carotenoid biosynthesis genes) is consistent with a transcriptional effort to remodel the LHC, towards increased photoprotection, for example by production of DLHCs; expected in Fe-limited, macronutrient replete conditions [4].

**Fig 4:**
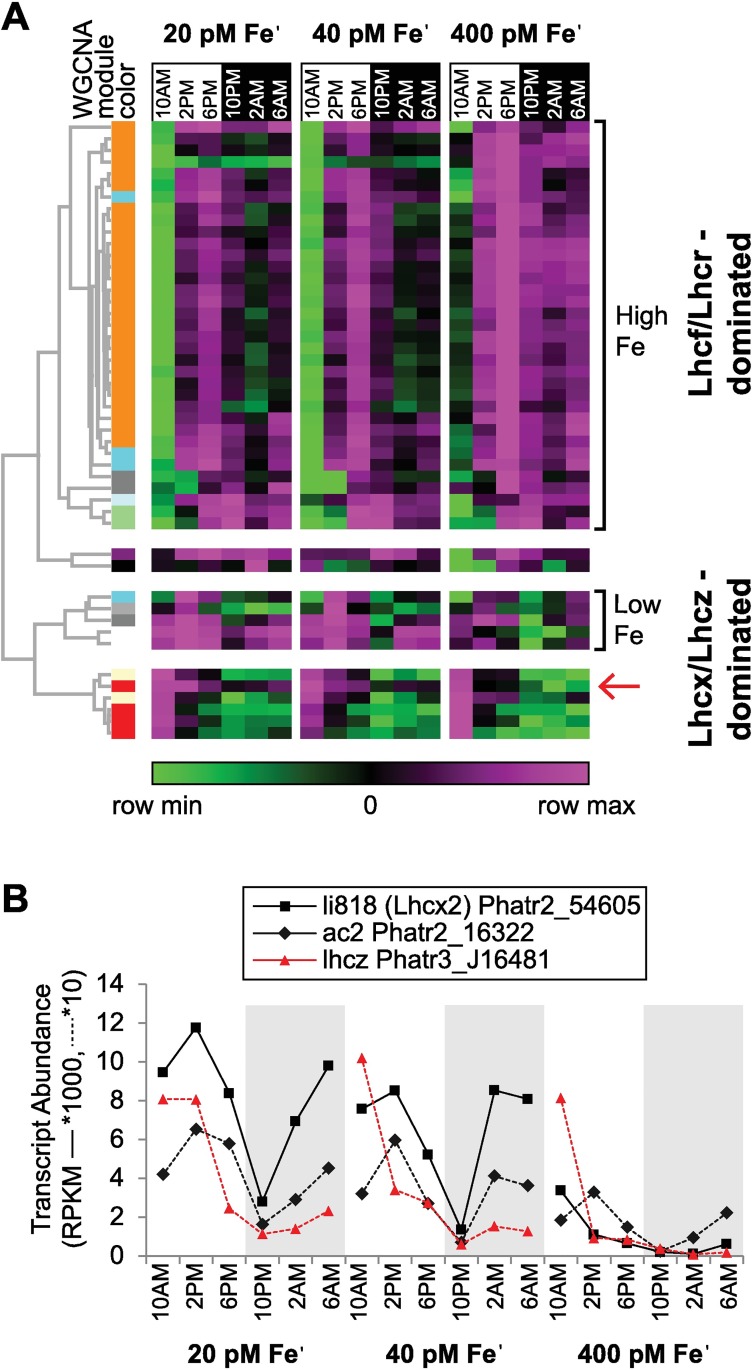
Light harvesting antenna protein expression. (**A**) Heat map shows min/max transcript abundance (RPKM across Fe conditions, plotted on a log scale). Dendogram shows hierarchical clustering (Pearson correlation) of RPKM values with corresponding module assignment from the WGCNA clustering analysis. Expression clades are labeled to indicate Fe-response types (high Fe, low Fe). Dominant protein family composition of expression clades (Lhcf/Lhcr and Lhcx/Lhcz) is also indicated. Gene identifiers (Phatr3 protein IDs) can be found in S3 Fig. (**B**) Selected expression patterns of Fe-sensitive light harvesting complex antenna proteins from the Lhcx, ac2, and Lhcz families. Lhcz (Phatr3_J16481) is from the red module and is indicated on the heat map with a red arrow.

The expression patterns of the full suite of LHC proteins identified in our dataset (n = 48) were analyzed to obtain a complete view of how these genes are modulated over diel cycles and in response to Fe. Phylogenetically, these proteins belong to 4 distinct groups: the chromophyte-specific fucoxantin Chl *a/c* LHCFs, the red algal-like LHCRs, LHCZ proteins and LHCX proteins [74]. Hierarchical clustering of the LHC transcript abundance patterns revealed that the majority are co-expressed (mirroring results obtained for the WGCNA module analysis) and belong to the LHCF/LHCR subtype (Fig 4, S11 Fig). Strong temporal dynamics were conserved across Fe conditions in this group. LHC transcript abundance was minimal directly following illumination (10AM) in all Fe conditions with peak mRNA detected in the late afternoon (Fig 4). LHC transcripts were also downregulated in *P*. *tricornutum* when illuminated following a 48hr dark period [75]. This suggests that cellular signals generated upon illumination are involved in repressing LHC transcription. It has been shown that this could be effected by PtCPF1, a cryptochrome/photolyase [76] which is maximally expressed in our experiments at 10AM. LHC expression is induced in the late afternoon, directly preceding the detectible onset of transcripts enriched for cell cycle progression in all Fe conditions (Fig 4). Expression of light harvesting genes has been coupled to chloroplast ontogeny at the G2/M transition in the diatom *Seminavis robusta*, and at S-phase in both *Thalassiosira pseudonana* and *P*. *tricornutum* [57,62,77]. Taken together our data further substantiates that transcriptional upregulation of the LHCs occurs at least in part as preparation for chloroplast replication during cell cycle division.

Genes of the photoprotective LHCX and LHCZ families had a different pattern of expression, with day-time maxima; the majority of which do not have any Fe-modulated expression (Fig 4). However, three LHC genes, from the LHCF, LHCZ, and LHCX families were significantly upregulated under low Fe (Fig 4B). Transcripts encoding two of these proteins (Lhcx2; Phatr2_54065, and Lhcz; Phatr3_16481) were previously shown to be Fe responsive [19]. LHCs, and especially members from the stress-response LHCX family, are thought to play an essential role in compensating for decreased levels of PSI units in Fe-deplete cells and to enhance non-photochemical quenching (NPQ) both under light and Fe stress [78–79]. A photoprotective function for LHCX1 (Phatr3_J27278) has been confirmed in *P*. *tricornutum* [79–80].

In addition to LHC remodeling, there are other changes to the light harvesting antenna that may have photoadaptive significance in Fe limiting conditions. For example, one of the most highly day-time induced transcripts at low Fe encoded a chloroplast-targeted heme oxygenase (Phatr3_J5902). Heme oxygenase catalyzes the rate limiting step in degradation of heme to bilirubin, producing ferrous Fe. In *Arabidopsis thaliana*, heme oxygenase is required to synthesize phytochrome photoreceptors [81]. It is possible that *P*. *tricornutum* heme oxygenase is involved in plastid Fe homeostasis, and may be upregulated to increase supply of ferrous Fe under these conditions, perhaps through recovery of Fe from damaged porphyrins. Further, membrane lipid desaturation in cyanobacteria lead to reductions in photoinhibition of PSII [82–84] and three genes encoding fatty acid desaturases showed a strong light-sensitive induction at low Fe in *P*. *tricornutum*, potentially as a strategy to protect the photosystem in a similar manner (Phatr3_J41570; Phatr3_J55137; Phatr3_EG02619).

### Diel oscillations in transcripts of primary metabolism

When illuminated, phototrophic organisms acquire light energy that is then stored within macromolecular chemical bonds. In the dark, stored chemical energy is then catabolized to meet cellular demands. To obtain a comprehensive view of how *P*. *tricornutum* regulates metabolic shifts between photoautotrophic (anabolic) and autoheterotrophic (catabolic) states, the diel expression of genes involved in several major primary metabolic pathways for acquiring, partitioning, storing, and catabolizing chemical energy was evaluated. A filtered set of 1,187 genes with either hypothesized or known direct or intersecting roles in central carbon metabolism and nitrogen assimilation were identified (S4 Dataset) and clustered hierarchically (methods). Strong, coordinated diel regulation of transcript abundance was observed for several essential genes in carbon metabolism. Broadly, pathways that were upregulated during the day included the Calvin Benson (CB) cycle, gluconeogenesis, carbohydrate biosynthesis, amino acid biosynthesis, mitochondrial glycolysis, fatty acid biosynthesis, and nitrogen assimilation. Nighttime expressed pathways included cytosolic and chloroplast glycolysis, carbohydrate degradation, the pentose phosphate pathway, fatty acid degradation (β-oxidation), and the TCA cycle.

**Fig 5:**
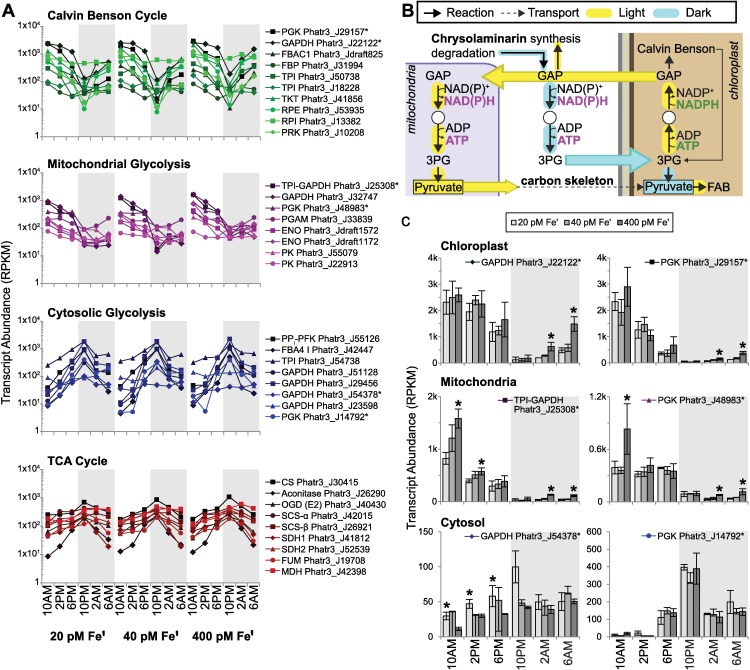
Coordinated expression of central carbon metabolic pathways. (**A**) Biological replicate averaged transcript abundance (for each time point) shown for genes from the Calvin Benson cycle, mitochondrial glycolysis pathway, cytosolic glycolysis pathway, and TCA cycle for each Fe condition (error bars omitted for clarity). Series labeled with gene names (see S4 Dataset for abbreviations) and Phatr3 PID. (**B**) Schematic representation of reactions catalyzed by GAPDH and PGK across the cytosol, mitochondria, and chloroplast (shown with 4 membranes). NADPH and ATP produced from light harvesting depicted in green, and NAD(P)H and ATP regenerated in the cytosol or mitochondria depicted in magenta. Yellow/blue highlighted arrows indicate day/night upregulation respectively. Solid arrows indicate likely transport processes, dashed arrow indicates hypothesized carbon skeleton transport. GAP: glyceraldehyde 3-phosphate, 3PG: 3-phosphoglycerate, FAB: fatty acid biosynthesis. (**C**) Transcript abundance for chloroplast, mitochondrial, and cytosolic targeted isoforms of glyceraldehyde phosphate dehydrogenase (GAPDH) and phosphoglycerate kinase (PGK) are plotted (TPI-GAPDH: triosephosphate isomerase-GAPDH fusion protein).

Genes in the CB cycle were among the most highly expressed metabolism-related transcripts in *P*. *tricornutum*, consistent with the central importance of carbon fixation for a photoautotroph (S12 Fig). Most CB transcripts reached peak expression at 10AM across Fe conditions, consistent with previous observations in *P*. *tricornutum* (Fig 5A) [85]. However, not all chloroplast-localized isoforms of putative CB enzymes peaked in expression during the light phase. Fructose bisphosphatase (FBP; Phatr3_J2793), phosphoribulokinase (PRK; Phatr3_EG02325), and a fructose/seduheptulose bisphosphatase (FBP/SBP; Phatr3_EG02409) were maximally expressed during the dark phase. Nocturnal expression indicates that these isozymes catalyze anapleurotic reactions, and are required during conditions in which the chloroplast is being resupplied with carbon skeletons in preparation for daytime carbon fixation. Co-expression of these genes and several putative chrysolaminarin genes with two triosephosphate transporters (TPT2; Phatr3_J46451, TPT4b; Phatr3_J24610), suggests that these transporters could mediate plastid carbon import under these conditions [86] (S13 Fig).

Carbohydrate biosynthesis genes (UGP-PGM; Phatr3_EG02613, SKN1; Phatr3_J50238, UGP; Phatr3_J23639, BGS1; Phatr3_J9617) were upregulated during the light when synthesis of the diatom storage carbohydrate chrysolaminarin occurs (S13B Fig) [87]. Conversely, putative chrysolaminarin and TCA cycle genes were upregulated at night reflecting elevated carbon catabolism (Fig 5A, S13B Fig). Photosynthetically fixed carbon is partitioned toward carbohydrates or the TCA cycle by glycolysis and gluconeogenesis, which are the central energetic and carbon metabolic pathways. Since many of the glycolysis/gluconeogenesis reactions are reversible, they are incompatible metabolic processes and in order for them to occur simultaneously they must be segregated either temporally (as in *Chlamydomonas)* or by intracellular compartmentation [30,88]. Diatoms possess several glycolysis/gluconeogenesis pathways localized to the chloroplast, cytosol, and notably (for stramenopiles) to the mitochondria [89–91]. The unique compartmentation of glycolysis in the mitochondria of diatoms (and other stramenopiles) has been identified previously, but the purpose of this organization is unknown; it does not exist in land plants or other eukaryotes [90–93]. Since this novel organization is characteristic of diatoms, diel expression of transcripts encoding these glycolytic/gluconeogenic enzymes was analyzed in order gain new insight into its significance.

Diatoms possess several isozymes for many steps in the cytosolic glycolysis/gluconeogenesis pathway and it remains unclear whether they are simply functionally redundant (i.e. extra copies) or whether they might be differentiated [90–91]. Some isozymes were more highly expressed during the day, while others were expressed at night indicating there could be more specialization than previously known. Specifically, two isozymes of GPI and FBA (GPI, Phatr3_EG02208 and FBA3 II, Phatr3_J29014) are co-expressed with the committed gluconeogenesis enzyme FBP (Phatr3_J23247). Selected carbon transporters (TPT1; Phatr3_J50742, TPT4a; Phatr3_J54017) were also diurnally co-expressed. TPT4a has been experimentally localized to the chloroplast inner membrane where it is hypothesized to play an important role in export or import of carbon to the cytosol [86]. Diurnal expression of these isozymes and their co-expression with carbohydrate biosynthesis is evidence that they may be specialized for plastid carbon export and gluconeogenesis. Other cytosolic glycolysis isozymes were most highly expressed in the dark phase and co-expressed with the TCA cycle, which is consistent with a role for this pathway in supplying pyruvate from catabolized carbohydrate for nocturnal respiration (Fig 5A). In contrast to cytosolic glycolysis, mitochondrial glycolysis isozymes were expressed maximally during the light phase and were coordinated with the CB cycle indicating that operation of this pathway may be elevated in the day, perhaps to directly process triose phosphates exported from the chloroplast (Fig 5). Though glycolysis/gluconeogenesis pathways were already known to be segregated spatially (compartmentalized) in diatoms, temporal segregation of the expression of these different glycolytic pathways into phases of the diel cycle is strong evidence that they are more dramatically functionally differentiated than previously understood.

An advantage of localizing glycolysis to the mitochondria and operating this pathway in the day is that diatoms can simultaneously synthesize carbohydrates (via cytosolic gluconeogenesis) and direct carbon towards the TCA cycle as pyruvate (mitochondrial glycolysis), processes which are otherwise incompatible if localized to the same compartment. While the actual fate of the pyruvate produced in mitochondrial glycolysis is unclear, it could either be exported from the mitochondria for biosynthetic processes in other compartments, or could enter the TCA cycle. The TCA cycle is both a degradative and biosynthetic, since its intermediates are drawn away as precursors for a wide variety of molecules such as amino acids. Based on the daytime expression of mitochondrial glycolysis and co-expression with other anabolic pathways, we hypothesize that this pathway may be biosynthetic and utilized to sustain daytime synthesis of amino acids and other important cellular molecules. A key consequence of mitochondrial glycolysis is that ATP and NAD(P)H fixed into GAP during the CB cycle are regenerated directly in the mitochondria, effectively shuttling ATP and NAD(P)H directly from the chloroplast to supply daytime mitochondrial energy demands (Fig 5B). Energetic coupling between diatom mitochondria and chloroplasts has been demonstrated, and *in silico* metabolic construction in *P*. *tricornutum* predicted that the nature of this connection is the mitochondrial glycolysis pathway [93, 94]. Our data validate a strong functional connection between CB and mitochondrial glycolysis, thus clarifying the metabolic significance of the latter pathway.

Few genes in these primary metabolic pathways were strongly Fe sensitive as defined by assignment to either the low Fe or high Fe response type (methods). Only one CB gene, fructose bisphosphate aldolase (FBAC5; Phatr3_J41423), previously identified to be highly sensitive to low Fe was upregulated in Fe limited cultures with high confidence [19,95]. However, some primary metabolism genes were more highly expressed at high Fe than in the more energy impoverished low Fe acclimated cultures. The CB cycle Ribose 5-P isomerase (RPI; Phatr3_J13382) and mitochondrial enolase (ENO; Phatr3_Jdraft1192) were both upregulated in high Fe-adapted *P*. *tricornutum* with high confidence. Other CB genes had elevated maximum transcript abundance in high Fe relative to low Fe at specific time points. For example, CB cycle transcripts of isoforms of GAPDH (Phatr3_J22122) and PGK (Phatr3_J29157) were significantly elevated at 2AM and 6AM in high Fe cultures (Fig 5C). Upregulation under high Fe reflects energetically replete carbon fixation conditions in *P*. *tricornutum* with unimpaired photosynthetic electron transport. Increased mRNA for the mitochondrial glycolysis genes TPI-GAPDH and PGK was observed in high Fe conditions at specific time points, similar to observations for the CB cycle (Fig 5C). At low Fe, *P*. *tricornutum* decreases expression of these genes, presumably to reduce the concentration of enzymes that partition photosynthetic energy to this pathway thus matching the reduced supply from impaired photosynthetic electron transport. Overall, this interpretation is consistent with physiological data showing that in diatoms with reduced growth rates, the proportion of the transient carbon pool belonging to cellular reductants is also reduced [96]. Cytosolic GAPDH and PGK transcripts were either elevated at or unaffected by low Fe (Fig 5C). We interpret the modest upregulation of cytosolic glycolysis in Fe limited cells as an attempt to increase cellular energy levels by drawing more carbon through this pathway to sustain production of ATP and NAD(P)H in the cytosol, and supply the TCA cycle and oxidative phosphorylation at night.

This pathway-level analysis of carbon metabolism across Fe conditions clarifies the functional differentiation between glycolytic pathways targeted to different compartments in *P*. *tricornutum*, and reveals that transcript abundance is modulated in a manner reflecting energy status at the different Fe conditions. At high Fe, when cells are energetically replete, there is an increased expression of genes involved in generating chemical energy, through carbon acquisition and storage in the light phase. These are some of the only genes in the *P*. *tricornutum* genome that are upregulated at high Fe along with genes of the light harvesting antenna and ribosomal proteins. At low Fe, *P*. *tricornutum* increases the expression of genes involved in acquiring energy from intracellular carbon (cytosolic glycolysis during the dark phase).

### Dark phase proteolysis and catabolism in *P*. *tricornutum*

The night appears to be a particularly active phase for cellular processes and metabolism, especially in low Fe cultures (Table 2). As discussed previously, dark expression of Fe acquisition genes, vesicle transport and autophagy genes, and partial synchronization of division with the dark phase means that a significant proportion of the differentially expressed transcriptome is involved in these processes. Additionally, the dark phase is particularly important for autotrophs since energy demands must be met by catabolizing macromolecules stored during the daytime. Reduced cellular nutritional quotas for *P*. *tricornutum* cultivated at low Fe would likely affect the magnitude of nighttime catabolic processes. To explore this, we analyzed diel fluctuations in the metabolite content and composition of *P*. *tricornutum* along with the functional roles of metabolic genes active in the nighttime transcriptome, and particularly those that were upregulated at the transcript level under low Fe.

**Fig 6:**
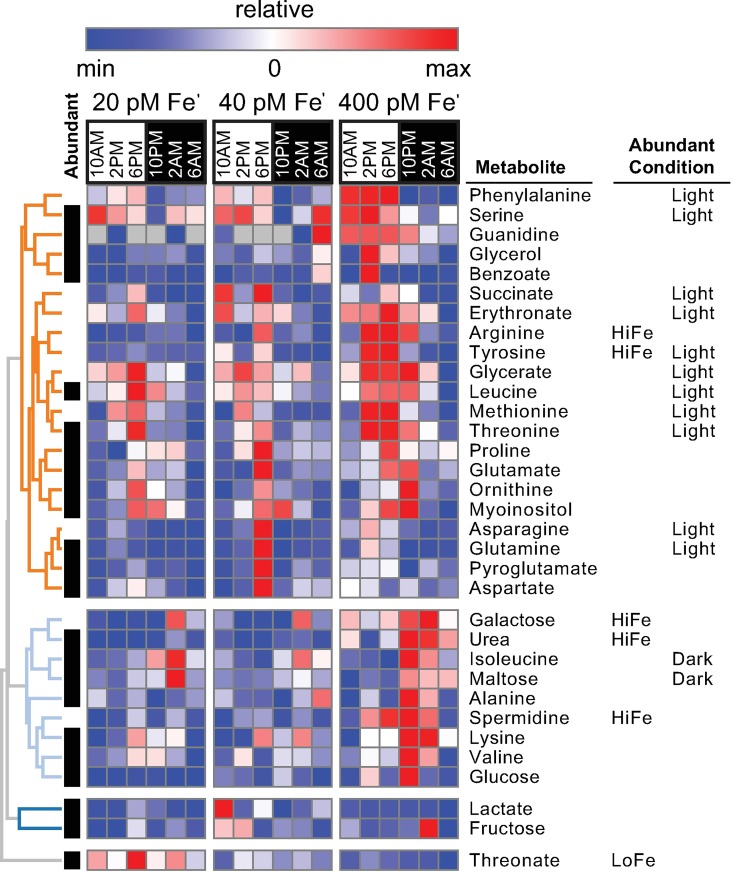
Fluctuations in metabolite composition of *P*. *tricornutum*. Abundant and/or condition-modulated metabolites are shown. Heat map colors scale to metabolite relative minimum and maximum across Fe conditions. Data are clustered hierarchically (Pearson correlation). Abundant metabolites (upper 50th percentile of average relative abundance) are indicated with black bars. Metabolites significantly more abundant at high Fe/low Fe are indicated (High Fe: 400pM Fe′, Low Fe: 20 & 40 pM Fe′ combined). Metabolites significantly more abundant during the light/dark phases are labeled (Light or Dark).

Diel fluctuations were documented for several metabolites (n = 33 total detected) with most reaching their maxima during the daytime (Fig 6). Generally, metabolites were more abundant in cells grown at 400 pM Fe′ than in cells grown at lower Fe concentrations, with only threonate elevated significantly at low Fe (Fig 6). Threonate is a product of ascorbate degradation, indicating that Fe limited *P*. *tricornutum* has higher ascorbate levels and may rely on its anti-oxidative properties to cope with elevated reactive oxygen stress [97–99]. Ascorbic acid likely functions in preventing photo-oxidative damage in Fe-deplete cells. On the other hand, if transported to the outer membrane, nighttime elevation of ascorbic acid can reflect an important role in the reduction of ferric to ferrous Fe during Fe-uptake; which has shown to be primarily segregated to the night phase in other phytoplankton and microbes [100–102].

Daytime metabolite composition was dominated by amino acids, but also included glycerate (and glycerate 3-P) formed in the first step of carbon fixation. Detection of these metabolites during the day is in agreement with general upregulation of essential transcripts for amino acid biosynthesis (S4 Dataset). At night, the metabolite composition of *P*. *tricornutum* shifts. The relative abundance of sugars (maltose, galactose) increases and is correlated with increases in mRNA for chrysolaminarin degradation genes, suggesting the observed accumulation is a result of increased catabolism of storage carbon. Urea and spermidine are also abundant at night, as are lysine and the branched chain amino acid isoleucine.

The global transcriptome was examined to identify metabolic drivers of these diel and Fe-specific metabolite shifts in *P*. *tricornutum*. Co-expression modules (lightcyan, royal blue) were identified which were strongly enriched with genes involved in proteolysis (S2 Table). Proteolysis may remediate the damage caused by elevated levels of ROS generated by an Fe-impoverished electron-transport chain [19]. Additionally, proteolysis could be upregulated in order to recycle fixed carbon and nitrogen to decrease dependence on extracellular input and the subsequent demand for additional reductant [19–20,103]. However, induction of genes in the lightcyan and royal blue modules was dark phase specific and conserved across Fe conditions, suggesting an Fe-independent function of this gene repertoire in the remodeling of a day-time to a night-time proteome, which could further serve as a dark phase source of amino acids.

**Fig 7:**
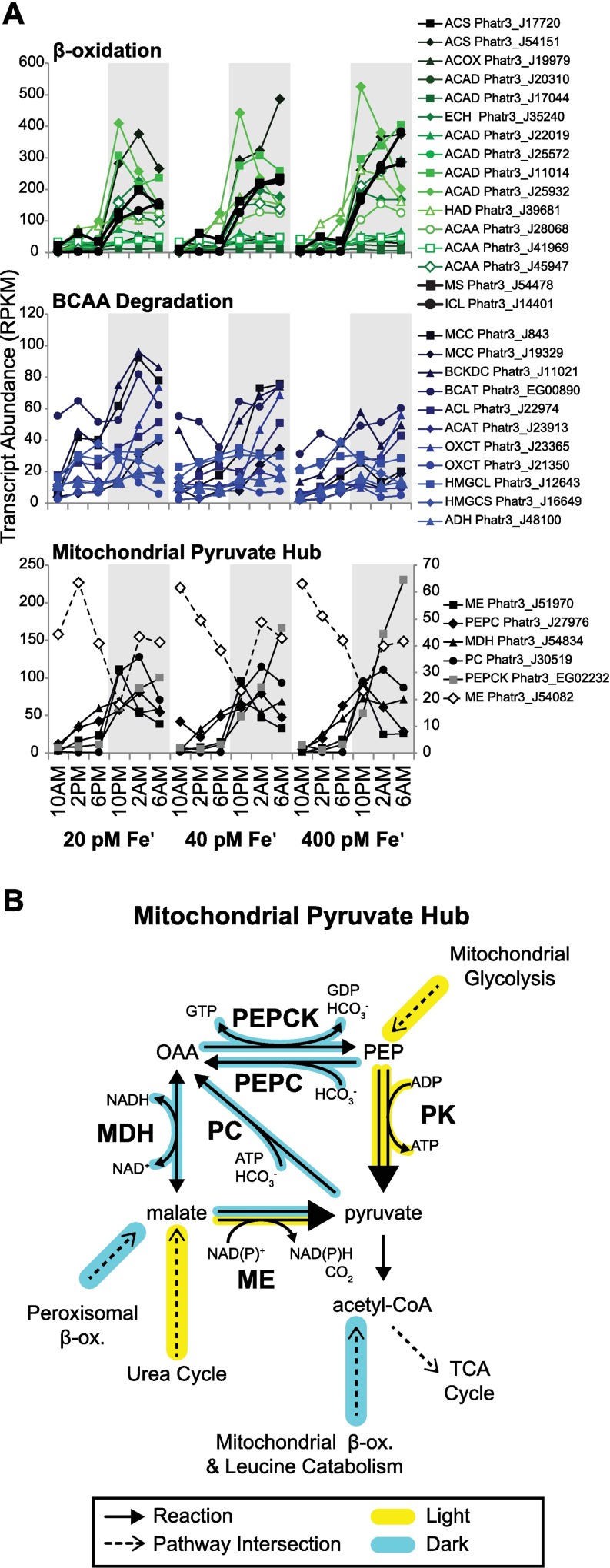
Dark phase expression of catabolism and pyruvate hub enzymes. (**A**) Biological replicate-averaged transcript abundance (for each time point) shown for genes from the β-oxidation (mitochondrial and peroxisomal combined), glyoxylate cycle, branched chain amino acid (BCAA) degradation pathways and mitochondrial pyruvate hub (error bars omitted for clarity). Series labeled with gene names (see S4 Dataset for abbreviations) and Phatr3 PID. (**B**) Pathway diagram showing reactions and co-reactants (H^+^ omitted) of the pyruvate hub. Each arrow represents a unique isozyme. PEP = phosphoenolpyruvate, OAA = oxaloacetate, PK = pyruvate kinase, ME = malic enzyme, MDH = malate dehydrogenase, PEPCK = phosphoenolpyruvate carboxykinase, PEPC = phosphoenolpyruvate carboxylase.

In addition to proteome remodeling, dark phase regulation of metabolic pathways likely affected metabolite composition. Several catabolic pathways are upregulated at night in *P*. *tricornutum* with varying degrees of Fe sensitivity. Fatty acid breakdown occurs through β-oxidation in the peroxisome and mitochondria to produce acetyl-CoA and propionyl-CoA that fuels nighttime respiration. Peroxisomal production of acetyl-coA is processed through the glyoxylate cycle prior to import into to the mitochondria as malate. Both β-oxidation and the glyoxylate cycle were strongly induced at night, and did not display sensitivity to Fe status (Fig 7). However, some transcripts encoding dark catabolism genes were highly upregulated in low Fe conditions, such as both isozymes of methylcrotonyl-CoA carboxylase (MCC1; Phatr3_J843, MCC2; Phatr3_J19329), and branched chain alpha-keto acid dehydrogenase E1, beta subunit (BCKDC, Phatr3_J11021), which are important for degradation of branched chain amino acids (BCAA; Fig 7). Recently, MCC1 has been shown to have a role in lipid metabolism [104]. Seemingly, *P*. *tricornutum* increases the capacity to catabolize a different pool of metabolites (BCAA) under low Fe compared to Fe replete conditions. Upregulation of BCAA catabolism in low Fe-adapted *P*. *tricornutum* could be in response to elevated influx of BCAA from the enhanced proteolysis occurring under these conditions. Beyond this, it is possible that the upregulation of BCAA catabolism is a response to having reduced carbohydrate and lipid stores under Fe limitation, forcing cells to catabolize less labile sources of carbon skeletons and chemical energy. Interestingly, acetolactate synthase (ALS; Phatr3_J37341), the first step in BCAA synthesis, was upregulated under low Fe, though it was maximally expressed during the daytime/anabolic phase. Apparently, under low Fe, *P*. *tricornutum* not only increases degradation of BCAA, but also increases the synthesis of these molecules, indicating there is a regulated shift in amino acid composition with some adaptive significance under low Fe conditions. The advantage of shifting towards enhanced BCAA metabolism in response low Fe remains unclear.

Carbon skeletons catabolically generated in the dark phase enter mitochondrial metabolism as a variety of 3C/4C molecules that intersect with the TCA cycle at different points (Fig 7B). The fate of 3C/4C skeletons may be determined by the activity of TCA enzymes as well as several other enzymes that catalyze intersecting reactions. Such enzymes include malic enzyme (ME), pyruvate carboxylase (PC), phospho*enol*pyruvate carboxykinase (PEPCK), and phosphoenolpyruvate carboxylase (PEPC), together referred to as the pyruvate hub [91,105]. Though these enzymes likely have important roles in cellular metabolism, though their specific functions remain largely unknown [90–91,105–108]. Most of the genes in the mitochondrial pyruvate hub are strongly upregulated in the dark, consistent with enhanced processing of 3C/4C skeletons during this phase (Fig 7). Perhaps this is to regulate metabolite abundance and flux into other intersecting pathways as a function of the availability of cofactors required for these reactions, such as NAD(P)H, ATP, and GTP (Fig 7). Notably, one of the mitochondrial isoforms of malic enzyme (ME; Phatr3_J54082) is strongly upregulated in the day, in stark contrast to the other pyruvate hub enzymes, suggesting it may have a daytime role in processing malate uncoupled from dark production in the glyoxylate cycle (Fig 7).

Due to the central importance of the TCA cycle in cellular energy generation, it is generally subject to post-transcriptional (i.e. allosteric, substrate supply, redox, post-translational) rather than transcriptional (coarse) regulation. Despite this, there were marked day: night fluctuations that were consistent within the greater catabolic context occurring in *P*. *tricornutum* in the dark phase, and indicate there is a clear component of transcript level regulation in facilitating metabolic flux shifts between illuminated and non-illuminated states.

### Diel regulation of nitrogen assimilation and the ornithine-urea cycle

**Fig 8:**
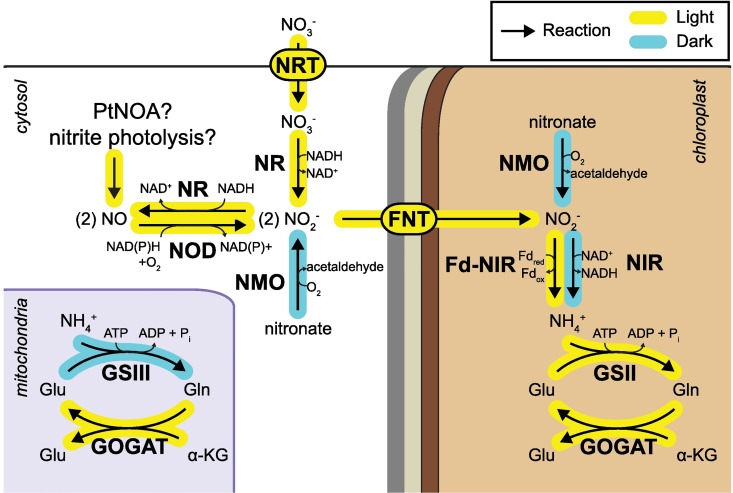
Diel expression of the N-assimilation pathway. Diagram shows the N-assimilation pathway and enzyme subcellular localizations with the general pattern of expression of genes indicated. Compartments are labeled, and the chloroplast is shown with four membranes. NRT = nitrate transporter, NR = nitrate reductase, NOD = nitric oxide dioxygenase, FNT = formate-nitrite transporter, Fd-NIR = ferredoxin-dependent nitrite reductase, NIR = NADH-dependent nitrite reductase, GS(II/III) = glutamine synthetase, GOGAT = glutamine oxoglutarate aminotransferase, NMO = nitronate monooxygenase. See S6 Fig for PIDs and normalized transcript abundance.

The acquisition of nitrogen is an essential process for maintenance and growth in phytoplankton, and nitrogen assimilation genes are some of the most highly expressed metabolism-related genes in *P*. *tricornutum* (S12 Fig). Nitrogen assimilation, particularly from nitrate, is energetically expensive requiring ATP and NAD(P)H [109]. Additionally, several steps in the pathway have a direct requirement for Fe. None of the genes involved in primary nitrogen assimilation were found to be sensitive to Fe at the transcript level statistically, but strong diel regulation of several key genes involved in primary nitrogen assimilation was observed (Fig 8). The nitrate transporter (NRT; Phatr3_J26029), nitrate reductase (NR; Phatr3_54983), formate/nitrite transporter (FNT; Phatr3_J13076), and chloroplast-localized Fd-NIR (Phatr3_J12092), were all coordinately expressed with maximum transcript abundance detected at 10AM (turquoise module, S14 Fig). Additionally, chloroplast-localized GS/GOGAT (GSII; Phatr3_J, GOGATchl; Phatr3_J24739) and mitochondrial GOGAT (GOGAT; Phatr3_J20342) were upregulated in the same way as NRT, NR, FNT, and Fd-NIR, though notably, mitochondrial GS (GSIII; Phatr3_J22357) was not coordinately expressed with these genes pointing to a different role in N metabolism for this enzyme (S14 Fig). The lack of coordinated expression of GSIII with other nitrogen assimilation genes has been previously observed in *T*. *pseudonana*, where GSIII was also found to be upregulated at night [110]. Also in low Fe cultivated *T*. *oceanica*, GS was found to be coordinately upregulated with isocitrate lyase (from the glyoxylate cycle), which is consistent with our observations [20]. With the exception of GSIII, there was coordinate, diurnal expression of the complete pathway for assimilation of nitrate to glutamine in *P*. *tricornutum*, expressed independently of Fe availability. We did not observe the downregulation in nitrogen assimilation transcripts at low Fe observed by Allen et al. [19] in Fe starved *P*. *tricornutum*. We attribute this difference to the fact that in the diel experiments, *P*. *tricornutum* growth was in steady state, indicating that expression of N assimilation transcripts is more affected by growth state than by Fe status alone.

Organic nitrogen is obtained through reduction of nitrate, but it can also be re-assimilated from nitric oxide. Nitric oxide is an important part of stress surveillance in diatoms, and a candidate gene involved in production of nitric oxide, nitric oxide synthase (PtNOA, Phatr3_J40200), has been identified in *P*. *tricornutum* [111–112]. In addition to production by PtNOA, nitric oxide can also be produced by abiotic photolysis of nitrite, or NR acting on nitrite as a substrate instead of nitrate [113]. How and when *P*. *tricornutum* would produce nitric oxide via NR is unclear, but this activity of NR may be either a mechanism to produce this signaling molecule under certain cellular nitrogen and energetic conditions, a wasteful process, or both. Nitric oxide can be recovered to nitrite by nitric oxide dioxygenase (NOD; Phatr3_J45621), which we identify here in *P*. *tricornutum*. Over diel cycles, PtNOA, NR and NOD were all co-expressed with primary nitrogen assimilation genes, supporting active turnover of the nitric oxide pool in coordination with the nitrogen assimilation pathway (S14 Fig). Specifically, this co-expression suggests that the relative activities of these enzymes (particularly NOD and NR) may function together in a system to regulate intracellular levels of nitrate, nitrite, and nitric oxide, and subsequently the magnitude of either N assimilation or NO production.

In marked contrast to most of the other nitrogen assimilation genes, chloroplast NADH-dependent nitrite reductase (NAD(P)H-NIR; Phatr3_EG02286) was upregulated during the dark (Fig 8, S14 Fig). NAD(P)H-NIR may sustain assimilation of nitrate during the dark phase, which has been observed to occur in diatoms [114–115], when the supply of reduced ferredoxin (from PET) is diminished in the dark. In addition to nitrite produced from nitrate (by NR) and from nitric oxide (by NOD), nitrite can be produced from the oxygenation of alkyl nitronates by nitronate monooxygenase (NMO). Two genes coding putative NMOs were identified in the *P*. *tricornutum* genome (Phatr3_J38755, Phatr3_J50353), and like NAD(P)H-NIR, these genes were expressed maximally at night (Fig 8, S14 Fig). Taken together, these data suggest that diatom nitrogen assimilation during the day and night phases may rely on temporally partitioned pathways and different nitrogen sources (day = nitrate/nitric oxide, night = alkyl nitronates), expanding on previous observations of diurnal and nitrate-limited expression of nitrogen assimilation genes in diatoms [110,116]. Ammonia produced at night by NAD(P)H-NIR may be assimilated by existing GSII machinery in the chloroplast, or transported to the mitochondria and assimilated into glutamine by GSIII. Nocturnal upregulation of GSIII is consistent with a role in assimilating ammonia released from dark phase proteolysis and amino acid catabolism. *P*. *tricornutum* possesses several ammonia permeases [117], and increases in nocturnal expression of these transporters (Phatr3_J10881, Phatr3_Jdraft972, Phatr3_J13418, Phatr3_J51516, Phatr3_J1862, Phatr3_J27877) indicates enhanced dark phase intracellular ammonia transport, further supporting this interpretation.

Diatoms possess an ornithine-urea cycle (OUC), considered to be an important metabolic hub at the intersection of cellular carbon and nitrogen, which is thought to have conferred a competitive advantage on diatoms [118–119]. However, little is known regarding the specific role of the OUC across diverse physiological states. Recent work by Levering et al. [120] identified the existence of a chloroplast ornithine cycle, the function of which was hypothesized to exchange NAD(P)H and ATP between the chloroplast and mitochondria. To gain insight into the biological role of these pathways and potential interactions between them, the diel and Fe-specific expression of genes and key metabolites involved in the OUC cycle were analyzed.

**Fig 9:**
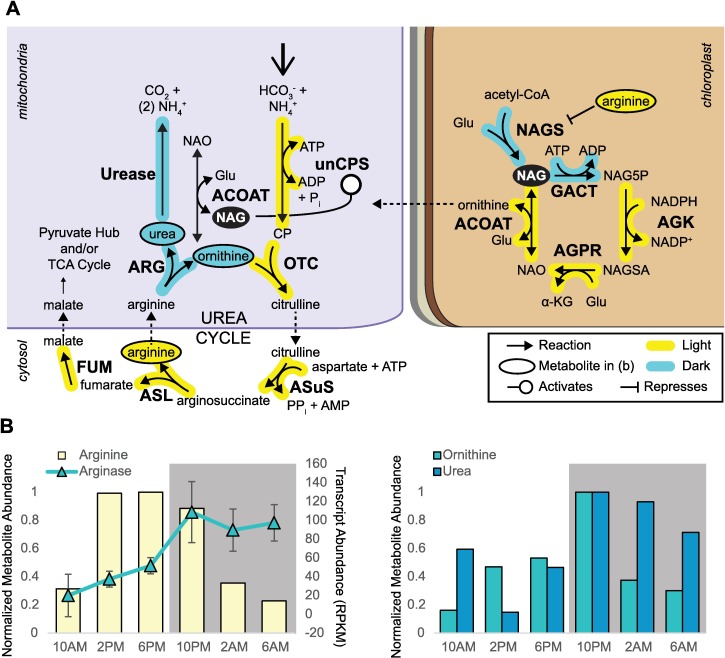
Urea cycle and chloroplast ornithine cycle expression and metabolites. (**A**) Pathway diagram shows the localization of reactions in the urea cycle, distributed between the mitochondria and the cytosol, and the chloroplast ornithine cycle [116,119]. Compartments are labeled, and the chloroplast is shown with four membranes. Dashed arrow indicates hypothesized transport. unCPS = carbamoyl-phosphate synthase, OTC = ornithine transcarbamylase, ASuS = arginosuccinate synthase, ASL = arginosuccinate lyase, FUM = fumarase, ARG = arginase, ACOAT = acetylornithine aminotransferase, NAGS = N-acetyl glutamate synthase, GACT = glutamate acetyltranferase, AGK = acetylglutamate kinase, AGPR = acetyl-glutamyl phosphate reductase. (**B**) Metabolite abundance (replicate averaged) over diel cycles in iron-replete conditions, shown normalized to maximum value within each metabolite. Average transcript abundance (across Fe conditions) for arginase is also plotted (with standard deviation).

Most of the core genes comprising the first four reactions of the urea cycle (unCPS; Phatr3_J24195, OTC; Phatr3_J30514, ASuS; Phatr3_J21116, ASL; Phatr3_J34526) were coordinately regulated with maximal expression detected during the light phase (Fig 9, S14 Fig). Arginase (ARG; Phatr3_J38509), however, which catalyzes the final step in the urea cycle was the exception and was most strongly expressed during the dark phase (Fig 9). The first four reactions of the urea cycle work together to synthesize the amino acid arginine, which accumulated during the day, and to much higher levels in *P*. *tricornutum* grown at 400 pm Fe′ (Fig 6 and Fig 9). Arginase then catalyzes the formation of ornithine and urea from arginine. Elevated arginase expression was indeed accompanied by a decrease in arginine and concomitant increase in both ornithine and urea, implicating this enzyme in modulating the relative abundance of these metabolites over diel cycles and under balanced growth (Fig 9). It is not clear why the complete urea cycle is not coordinately expressed, but it is consistent with earlier observations of urea cycle expression in *P*. *tricornutum* grown on alternate N sources [118], in which arginase transcript abundance did not correlate with expression of the other urea cycle enzymes. We hypothesize that the urea cycle functions primarily in the day to synthesize arginine, and that the fumarate produced in this process is returned to the TCA cycle as malate (Fig 7 and Fig 9). To sustain arginine biosynthesis, an input of ornithine to the mitochondria in the absence of arginase activity is required. Considering the pattern of urea cycle expression and metabolic shifts observed over day: night cycles, we propose a conceptual model in which the chloroplast ornithine cycle and urea cycle are coordinated to regulate metabolite shifts based on cellular energy, carbon, and nitrogen status (Fig 9).

Unlike the urea cycle, the chloroplast ornithine cycle is capable of synthesizing ornithine *de novo* with N-acetylglutamate synthase (NAGS) catalyzing the first step in which glutamate and acetyl-CoA are utilized to synthesize N-acetylglutamate (NAG). Two putative NAGS were identified in the *P*. *tricornutum* genome, which lacked high confidence plastid-targeting predictions but still may have N-terminal targeting sequences (NAGS1; Phatr3_J10902, NAGS2; Phatr3_J44492, S15 Fig). Under conditions in the chloroplast when glutamate, acetyl-CoA, NADPH, and ATP are elevated (i.e. illuminated, N-replete), ornithine production is favored [120]. Under this scenario, ornithine is then exported from the chloroplast, supplying the urea cycle for arginine biosynthesis, and effectively signaling to the mitochondria that the chloroplast is metabolically “charged”. In bacteria and unicellular eukaryotes, there is negative feedback of arginine levels on NAGS activity, thereby modulating arginine biosynthesis [121]. The NAGS isozymes that *P*. *tricornutum* possesses are most similar to sequences from other diatoms, red algae, and oomycetes, suggesting that they were acquired during the secondary endosymbiosis and supporting the idea that they could be regulated by the arginine feedback inhibition characteristic of NAGS from these lineages [121]. Simultaneously in the mitochondria, the first phase of the urea cycle is favored towards arginine biosynthesis when NH_4_^+^, HCO_3_^-^, aspartate, and ATP are abundant, and there is a reliable supply of ornithine imported from the chloroplast, which again occurs during energetically favorable and N-replete conditions. Abundant glutamate favors the production of NAG in the mitochondria (by ACCOAT). In humans, NAG activates the urea cycle by allosterically interacting with CPSI, in order to signal urea production and excretion at high cellular nitrogen levels [121]. Phylogenetically, diatom mitochondrial CPS (unCPS) clades with human CPSI [118]; therefore, it is likely that the form found in diatoms (unCPS) is also activated by NAG. However, the ultimate outcome in diatoms would be to favor arginine biosynthesis at high nitrogen, rather than urea production. In the dark, conditions for arginine biosynthesis are no longer favorable, and arginase is upregulated, converting arginine synthesized in the daytime to ornithine and urea. An increase in mitochondrial ornithine decreases NAG production, slowing the urea cycle and reducing the flux of precursors towards the arginine biosynthesis pathway. Urease (Phatr3_J29702) expression is strongly upregulated at night, correlating with a decrease in cellular urea levels, and increasing the nocturnal supply of nitrogen in the form of ammonia. Other genes involved in the OUC, such as agmatinase (Phatr3_ J40880), ornithine cyclodeaminase (Phatr3_J35643), and ornithine aminotransferase (OAT; Phatr3_J27726), were also upregulated at night.

This model of nitrogen metabolism explains mechanistically why at low Fe there are statistically significant decreases in metabolites including arginine and urea (Fig 7). *P*. *tricornutum* is not limited for N at any of the Fe′ concentrations examined, but the biosynthesis of arginine and urea rely on availability of carbon skeletons (from acetyl-CoA), and sufficient ATP and NAD(P)H, which have been seen to be limiting under low Fe acclimated *P*. *tricornutum*. Further, this model highlights the degree to which the chloroplast and mitochondria are metabolically and energetically connected, and able to communicate their metabolic status through the dynamic exchange of carbon skeletons and nitrogen groups. Despite the essential roles of transporters in regulating pathway connectivity and intracellular metabolite fluxes, these transporters remain conspicuously absent from this and many models of diatom metabolism [90–92,108,120]. In the *P*. *tricornutum* genome, there are over 350 putative transporters (intracellular and extracellular) that are dynamically expressed over day: night cycles (S16 Fig). The majority of transporters are induced in the dark phase, perhaps to accommodate elevated shuttling of metabolites within cellular compartments during enhanced division, proteolysis, autophagy, and catabolism observed at this time. Effective interpretation of the significance of fluctuating transporter transcripts requires better biochemical characterization and localization of these transporters. Such knowledge will be essential for construction of accurate metabolic models, required to further improve our understanding of the significance of fluctuating transcripts over diel cycles.

## Conclusions

Oceanic phytoplankton must possess adaptations that confer survivability in adverse conditions, and strategies used to cope with varying levels of resource availability provide insight into forces that structure phytoplankton populations across different ocean regimes. Initial molecular insights into adaptations diatoms possess to cope with limiting Fe, such as Fe-acquisition machinery, have begun to explain why this group is so successful in Fe-limited regions of the ocean, but have generally not been studied in a dynamic physiological context. In addition to nutrient acquisition, primary producers must cope with vast fluctuations in energy inputs across day: night cycles. Mechanistically, how phytoplankton are able maintain homeostasis and coordinate growth under these dramatic metabolic and energetic shifts is just beginning to be understood in detail.

*P*. *tricornutum* exhibits massive and coordinated shifts in the global transcriptome, metabolome, and physiology over day: night cycles revealing the extent to which fluctuations in light drive major cellular changes. Through incorporating time course expression measurements of Fe-sensitive genes, we identify the night as a particularly important phase for Fe metabolism in both Fe-limited and Fe-replete cultures. At night, Fe-limited cultures also express genes associated with catabolism, proteolysis, autophagy, and endocytosis more highly than Fe-replete cultures, suggesting that efficient recycling of intracellular Fe and acquisition of Fe from the environment are essential for successful acclimation to low Fe. Functional investigation of this highly expanded Fe-sensitive gene catalog, including genes involved in endocytosis and gene regulation (i.e. transcription factors) will be important to further characterize the specific mechanisms diatoms possess to acquire, respond and acclimate to low Fe.

Diel expression of core metabolism reveals the strong influence of light:dark cycles in regulating primary metabolism at the transcriptional level. Expression of these metabolic pathways was largely unaffected by Fe-limitation. However subtle shifts in metabolism, such as the upregulation of cytosolic glycolysis and branched chain amino acid metabolism function to maintain the metabolic and energetic homeostasis required to support growth in low Fe cells. Downregulation of energy acquisition (light harvesting and carbon storage), reduction in growth rate, and upregulation of Fe acquisition appear to work together to “protect” core metabolic function across diel cycles, revealing the metabolic resilience of *P*. *tricornutum* to low Fe. These insights explain further how diatoms persist in Fe-limited areas of the global ocean, and highlight specific adaptations which may have contributed to their ecological dominance in these regions.

## Methods

### Cultivation and physiological measurements

Steady-state cultures of axenic *P*. *tricornutum* (CCMP2561) were maintained under a diel cycle of 12h Light: 12h darkness (320 μmol photons·m^−2^·s^−1^; lights ON at 9 AM; lights OFF at 9PM) at 18°C and at three different concentrations of Fe′ (Fe′: sum of all Fe species not complexed to EDTA): a low concentration (20 pM Fe′), intermediate concentration (40 pM Fe′) and at Fe-replete levels (400 pM Fe′). Cultures were grown in a modification of Aquil medium [122] containing 100, 10 and 100 **μ**M nitrate, phosphate and silicates, standard “f/2” vitamin concentrations, and 300 μM EDTA with free Zn, Mn, Co, Cu, MoO_4_ and SeO_3_ concentrations (expressed as negative logarithms) of 10.93, 8.03, 10.77, 12.63, 7.00 and 8.00. Total Fe concentrations added were 15, 30 and 600 nM Fe. Fe′ was calculated assuming a 2 nM background Fe in our Aquil preparation, and the equations in Sunda et al. [122] considering temperature, pH and diel-integrated light levels. Cells were acclimated to each Fe condition in 250 mL polycarbonate bottles, and growth rates were calculated as the natural log of cell density over time, using a Beckman-Coulter counter. After growth rates changed by less than 10% in subsequent transfers (usually by 30 generations), 100–200 mL of each culture was inoculated into 20 L cultures, and growth was monitored as before. At each time point, 250 mL of cultures was harvested at an average density of 4.84x10^5^ cells mL^-1^ (sd = 1.15x10^5^ cells mL^-1^), and fresh (and equilibrated with respect to metal speciation) media was added to maintain modest cell densities throughout the diel sampling period. Samples were taken three times during the day (10AM, 2PM, 6PM) and during the night (10PM, 2AM, 6AM). Three distinct diel experiments were run, including one with high Fe conditions only and an additional two with all three experimental treatments, leading to independent duplicate diel data sets for the 20 and 40 pM Fe′ treatments and triplicate diel data sets for the 400 pM Fe′ treatment, totaling 49 samples (S1 Table). Growth curves for each experiment are included (S1 Fig–S7 Fig). Due to a computational correction, the Fe′ values should be 27, 54, and 540 pM instead of 20, 40, and 400 pM.

At each time point, triplicate determinations of F_v_/F_m_ were made using the DCMU method [123]. The significance of nocturnal drop in F_v_/F_m_ value was tested by a paired t-test of the diurnal maxima vs. the nocturnal minima in either low Fe conditions or Fe-replete conditions.

Percentage dividing cells was microscopically determined by observations of culture samples using a Zeiss Axioplan 200 equipped with differential interference contrast (DIC) module. Therefore, part of the sample at each time point was fixed with formaldehyde at 0.5% final concentration using freshly prepared paraformaldehyde in PBS. Discrimination of dividing vs. interphase cells was achieved at 600–1000× total magnification, based on the quantification of doublet cells. Doublet cells characterized by a daughter cell wall that is only present in mitotic cells and for each sample a minimum number of 200 cells were observed. This quantification resulted in a conservative estimation of M-phase cells, since only part of the M-phase (actual cytokinesis), was considered, thereby excluding nuclear division during karyokinesis.

### RNA extraction and sequencing

During sampling, an average of 1.21 × 10^8^ cells ml^-1^ was harvested (250 mL) on 0.45 **μ**m Sterivex filters and flash frozen in liquid nitrogen. Total RNA was extracted using Trizol reagent: (Thermo Fisher Scientific), enriched and amplified (15 cycles) in mRNA using MessageAmp II aRNA Amplification Kit (Ambion). 80 ng of amplified mRNA was used for SOLiD library construction using SOLiD Total RNA-Seq Kit (Applied Biosystems). SOLiD-based transcriptome sequencing of poly-A enriched mRNA was performed on all 49 experimental time points. Sequence data (n = 49 samples) were deposited and are available at the NCBI Sequence Read Archive (SRA) under the identifier SRA: SRP069841.

### Read-mapping and RPKM calculations

Paired-end SOLiD reads (50 bp forward and 35 bp reverse) were mapped to the *Phaeodactylum tricornutum* genome via Life Technologies LifeScope software (which handles reads in SOLiD color space format directly). This mapping yielded a BAM alignment, which, combined with a file detailing the genomic locations of each *P*. *tricornutum* exon, was converted to a set of read counts per exon using the samtools package. An in-house script was used to aggregate gene counts from exons and to compute RPKM values, where gene RPKM = gene_read_count / (total_mapped_reads (Mb) * gene_transcript_length (kb))

RNA-Seq reads for each of the 49 libraries were mapped to both the *P*. *tricornutum* Phatr2 assembly (ASM15095v2), which includes 34 finished chromosomes and 55 unassembled scaffolds [94] and the re-annotation of the *P*. *tricornutum* genome (Phatr3) (http://protists.ensembl.org/Phaeodactylum_tricornutum/). The re-annotation effort was augmented by 90 new RNAseq libraries, in addition to the 49 considered in this study, leading to the discovery of additional gene models and improvement of gene model boundaries. Quantitative temporal transcript levels were calculated for both versions of the *P*. *tricornutum* genome, yielding RPKM values for 10,577 gene models from Phatr2 and 12,294 gene models from Phatr3. These totals include transcript levels for genes on the chloroplast and mitochondrial genomes (n = 160). Though generally gene models were improved in Phatr3, reads were mapped back to both versions of the gene models to obtain transcript levels for gene models that were erroneously deleted or called incorrectly in Phatr3. A comparison between Phatr2 and the updated Phatr3 gene models, and RPKMs for version of *P*. *tricornutum* gene models are included in S5 Dataset.

Subsequent genome-scale analyses utilized the transcriptome mapped to Phatr3 gene models, and all reported gene expression is from Phatr3 except when specifically denoted. Significance of differences of mean vs. median RPKM ratios were verified using a Wilcoxon rank-sum test performed in Matlab using group-based ratios for all genes in the active transcriptome. This led to the identification of 10,588 genes in the *P*. *tricornutum* genome that were transcriptionally active under the tested experimental conditions. The number of Phatr3 genes which had an average count value greater than 10 and minimum count greater than 1 were scored, resulting in the identification of 10,557 genes which were considered transcriptionally active. Of these, 158 were genes from the mitochondrial and chloroplast genomes, meaning 10,399 nuclear genes were actively transcribed. In the manuscript, both RPKM and standardized RPKM [124] are reported.

### Global Expression Analysis

#### WGCNA clustering

Correlation network analysis of gene expression diel profiles was performed using the WGCNA package in R, following the principles laid out by Langfelder and Horvath. We specifically applied the comparative consensus network option to compare data sets across conditions [45]. The consensus network was constructed using the 20 pM Fe′ and the 400 pM Fe′ conditions, but to increase performance only data from most highly expressed nuclear genes (8,499 genes; Median ≥ 10 RPKM) were used as input. Application of a *signed* network was crucial to allow clustering of oppositely correlated gene expression values in distinct modules. In addition, by raising the absolute value of the pairwise gene expression correlations to the soft-thresholding parameter *β* = 30, the scale-free topology criterion for WGCNA was achieved, thereby emphasizing high correlations at the expense of low correlations [45]. Selection of *β* occurred as described by Langfelder and Horvath [45] through testing its dependence (5<*β* <40) on the scale-free topology fit model for both Fe conditions (S17 Fig). Thus, for construction of the consensus network, the function *blockwiseConsensusModules* was used using the following parameter settings: *β* = 30, *networkType* = "signed", *TOMType* = "signed". Subsequently, modules were detected on basis of the Topological Overlap Measure (TOM) using the following parameters: *minModuleSize* = 40, *deepSplit* = 2, *maxBlockSize* = 10000, *mergeCutHeight* = 0.20. These parameters resulted in detection of 27 color-coded modules that described distinct units of correlated gene expression in both Fe conditions. The color-code ‘grey’ ME is reserved for background genes that could not be clustered.

#### Response type binning

Genes with significant responses to Fe and illumination state were identified by binning replicate time points per Fe and light condition and applying the non-parametric Skillings-Mack (SM) test for 2-way experimental design in the TM4 gene expression software suite [125–129]. With significance threshold set at *P* ≤ 0.01, 55% percent of the active transcriptome (5,803 genes; 45% of the *P*. *tricornutum* genome) was sorted into four main expression groups (*P* ≤ 0.01). Genes were categorized as possessing higher transcript abundance in the dark (Dark) or in the light (Light), and at low Fe (20–40 pM Fe′) conditions or at high Fe (400 pM Fe′) relative to the opposite condition. The direction of Fe or light-dependent modulation, either an increase or decrease of transcript abundance in each condition, was identified by calculation of the log_2_ of the average RPKM ratio within the Low Fe vs. High Fe and Light vs. Dark samples, and is reported in S1 Dataset, S2 Dataset, S4 Dataset, and S5 Dataset as “Iron Depl. vs. Iron Repl” and “Light vs. Dark”.

The differential expression of genes responsive to Fe and illumination state was validated using EdgeR with a generalized linear model using the likelihood ratio test (LR) in a two factor analysis [130]. P-values were adjusted by the Benjamini-Hochberg procedure for multiple testing correction to control for false discovery rate at a level of adjusted p < 0.05. Response type bins for each gene are shown in S1 Dataset with genes found to be significant for the same response category by both methods called “high confidence”. The larger set of genes found to be significant by the SM test are considered the primary response type bins unless otherwise specified, including those that are high confidence. This approach to binning was chosen to represent a more inclusive set and was, in this dataset, found to be more consistent with genes known to be responsive to Fe. Among the iron starvation inducible protein (ISIP) genes, all were called significant in low Fe conditions by SM, but ISIP2a was not called significant by EdgeR.

#### GO functional enrichment calculations

Gene functional enrichments (FDR-corrected P ≤ 0.05) were identified in differentially modulated gene groups against the whole genome as a reference. Significance was assessed using a Binomial test and Benjamini & Hochberg's FDR multiple testing correction using the BiNGO Cytoscape plugin [131].

### Identification and curation of metabolic pathways

Genes were assigned to metabolic pathways and transport processes by manual curation in Phatr3, using a combination of approaches and criteria. First, genes were identified with keyword searches of both existing and *de novo* gene catalog annotations, and published pathway annotations specific to *P*. *tricornutum* [85–86,90–92,132]. To identify all desired isozymes of individual steps in desired pathways, we used BLASTP against the *P*. *tricornutum* genome (draft version at NCBI, Phatr2 version at JGI, and/or Phatr3 version at ENSEMBL) utilizing query sequences from validated genes from model organisms, or *P*. *tricornutum* isozymes. Identified genes were then curated and functionally assigned based on a variety of criteria. These criteria were the presence of specific conserved domains (protein queries to the NCBI Conserved Domain Database), subcellular bioinformatics-based targeting predictions, and expert knowledge of biochemistry and biochemical pathways. Subcellular targeting predictions were improved in Phatr3 with extensions to gene models to include full N-terminal ends where targeting peptides are found. When high-confidence subcellular localization predictions were important for pathway curation, these predictions were further refined by using SignalP 3.0 [133], SignalP 4.1 [134], ASAFind v1.0.0 [135], Mitoprot [136], and TMHMM [137].

### Metabolite analysis

Metabolites were extracted and derivatization and GC-MS analysis were carried out as described in Lisec et al. [138], with minor modifications made for diatom analysis as defined in Allen et al. [19]. Metabolites were identified through comparison with database entries of authentic MSRI libraries [139]. The impact of illumination or Fe availability on the abundance of 35 metabolites was determined using T-test (*P*-value < 0.05).

## Supporting Information

S1 Dataset Active transcriptome and assignment of genes to WGCNA modules and response types. (XLSX)

S2 Dataset List of highly low Fe responsive genes. (XLSX)

S3 Dataset List of low Fe induced genes involved in endocytosis, exocytosis and autophagy. (XLSX)

S4 Dataset Metabolic pathway annotations. (XLSX)

S5 Dataset Phatr3 and Phatr2 comparison. (XLSX)

S1 Fig Natural log of cell density versus time preceding and during experiment L1 at 20 pM Fe′. The start of the diel is indicated by filled inverted triangles, times of carboy inoculation used for diel is indicated by hollow triangles, and time is given as ordinal day 2010. In (**A**) and (**C**), breaks in regression lines indicate culture transfer (prior to diel sampling) or dilution (during diel sampling). (**A**) Cell density versus time seven days prior to and during the diel sampling period. (**B**) Cumulative cell density computed from initial starting density (disregarding dilution events) in order to calculate and visualize growth history prior to and during diel experiments. Growth rates, as the slope of the natural log of cell density are shown for the period preceding and after carboy inoculation. (**C**) Cell densities determined by coulter counting during the diel. Vertical lines indicate transition between light and dark phases. Cell densities were maintained from 400,000–530,000 cells mL^-1^ during the diel.
(AI) Click here for additional data file.

S2 Fig Natural log of cell density versus time preceding and during experiment L2 at 20 pM Fe′. As in S1 Fig, except in panel (**C**), cell densities were maintained from 400,000–526,000 cells mL^-1^ during the diel.
(AI) Click here for additional data file.

S3 Fig Natural log of cell density versus time preceding and during experiment M1 at 40 pM Fe′ As in S1 Fig, except in panel (**C**), cell densities were maintained from 415,000–840,000 cells mL^-1^ during the diel.
(AI) Click here for additional data file.

S4 Fig Natural log of cell density versus time preceding and during experiment M2 at 40 pM Fe′. As in S1 Fig except in panel (**C**), cell densities were maintained from 375,000–517,000 cells mL^-1^ during the diel.
(AI) Click here for additional data file.

S5 Fig Natural log of cell density versus time preceding and during experiment H1 at 400 pM Fe′. As in S1 Fig, except in panel (**C**), cell densities were maintained from 360,000–946,000 cells mL-1 during the diel.
(AI) Click here for additional data file.

S6 Fig Natural log of cell density versus time preceding and during experiment H3 at 400 pM Fe′. As in S1 Fig, except in panel (**C**), cell densities were maintained from cells mL-1 during the diel.
(AI) Click here for additional data file.

S7 Fig Natural log of cell density versus time preceding and during experiment H2 at 400 pM Fe′. As in S1 Fig, except in panel (**C**), cell densities were maintained from 340,000–540,000 cells mL^-1^ during the diel.
(AI) Click here for additional data file.

S8 Fig Expansion of low Fe sensitive gene catalog in P. tricornutum. Diel expression of low Fe response type genes (n = 2090, S2 Dataset, methods). Transcript abundance patterns are hierarchically clustered (Pearson correlation). Heat map shows standardized transcript abundance (RPKM across Fe conditions) for each gene. Black bars indicate which genes were identified as low Fe responsive by each statistical approach (Skillings-Mack vs. EdgeR), as well as genes previously identified as low Fe responsive in [19].
(AI) Click here for additional data file.

S9 Fig Transcription factor expression pattern. (**A**) Heat map shows standardized transcript abundance (across Fe conditions) for annotated transcription factors [54] clustered hierarchically (Pearson correlation). Adjacent heat maps indicate the maximum transcript detected for each gene across experiments (RPKM), and the degree to which genes responded to Fe (see methods). Names and PIDs of the top ten most highly expressed genes and the top ten most highly iron-responsive genes are labeled, with overlap between these lists noted. Predicted organellar localization is indicated. (**B**) Transcript abundance of CCHH11 is indicated, and shown RPKM values from each experimental time point (not averages). (**C**) Output from TMHMM showing a C-terminal transmembrane domain in the CCHH11 gene model (Phatr3_J38018).
(AI) Click here for additional data file.

S10 Fig Cyclin and CDK expression. Heat map shows transcript abundance (RPKM) for *P*. *tricornutum* cyclins and CDKs [62]. Genes binned as low Fe responsive are shown in bold (*Binned by Skillings-Mack, †Binned by EdgeR). Dendogram shows hierarchical clustering (Pearson correlation) of RPKM values. Gene ID, gene names, and relative expression level (max RPKM for each gene normalized to max across entire gene set) included to illustrate which cyclins/CDKs transcripts are most abundant. Cyclin and CDK transcripts previously assigned to cell cycle phases [62] are indicated.
(AI) Click here for additional data file.

S11 Fig Light harvesting antenna protein expression. Heat map shows transcript abundance (RPKM, plotted on a log scale). Dendogram shows hierarchical clustering (Pearson correlation) of RPKM values. Protein ID, gene names, WGCNA module and response type (SM: Skillings-Mack and EdgeR) are indicated for each gene.
(AI) Click here for additional data file.

S12 Fig Most highly expressed primary metabolic pathways. (**A**) Bar plot shows maximum and median RPKM detected for each gene across the entire experiment (all time points and Fe conditions) annotated with a primary metabolic function, organized by pathway (S4 Dataset). (**B**) List of the pathways included and the number and percentage of genes assigned to each pathway with a maximum RPKM greater than 500. (**C**) List of the genes within the most highly expressed pathways with a maximum RPKM greater than 500, with maximum and median values indicated. Genes binned as Fe-responsive (low Fe and high Fe) are indicated. *Binned by Skillings-Mack, †Binned by EdgeR
(AI) Click here for additional data file.

S13 Fig Carbohydrate biosynthesis and degradation. (**A**) Pathway diagram shows the putative localization of glycolysis, gluconeogenesis, and carbohydrate biosynthesis and degradation enzymes. Compartments are labeled, and the chloroplast is shown with four membranes. Each line connecting metabolites indicates an isozyme. (**B**) Biological replicate averaged transcript abundance (for each time point) shown for genes from gluconeogenesis, carbohydrate biosynthesis, carbohydrate degradation, and dark phase Calvin Benson cycle. Error bars omitted for clarity, series labeled with gene names (see S4 Dataset for abbreviations) and Phatr3 PID.
(AI) Click here for additional data file.

S14 Fig Expression patterns of nitrogen metabolism genes. Average-normalized transcript abundance for genes involved in nitrogen assimilation, dark phase nitrogen assimilation, urea cycle, and chloroplast ornithine cycle is shown. Series labeled with gene names (see S4 Dataset for abbreviations) and Phatr3 PID.
(AI) Click here for additional data file.

S15 Fig N-acetylglutamate synthase (NAGS) gene candidates. (**A**) Table summarizing targeting predictions (see methods) for both NAGS1 and NAGS2 gene candidates. (**B**) Sequences used for gene targeting, with Phatr3 gene model ID and EST-based N-term extensions (NAGS1) indicated. (**C**) Domain architecture (from NCBI blastp) showing hits to PRK05279 (N-acetylglutamate synthase, validated).
(PDF)

S16 Fig Transporter expression patterns. Heat map shows standardized transcript abundance (across Fe conditions) of all putative transporters clustered hierarchically (Pearson correlation, S4 Dataset). Adjacent heat maps indicate the maximum transcript detected for each gene across experiments, and the degree to which genes responded to Fe (see methods).
(AI) Click here for additional data file.

S17 Fig Scale-free topology model fit as function the soft thresholding power (x-axis). Numbers in the plots indicate the corresponding soft thresholding powers. The plot indicates that the approximate scale-free topology is attained around the soft-thresholding power of 30 for both Fe conditions.
(AI) Click here for additional data file.

S1 Table Experimental design. (PDF)

S2 Table GO term enrichment in WGCNA modules. (PDF)

## References

[1] Martin JH, Fitzwater SE. Iron deficiency limits phytoplankton growth in the north-east Pacific subarctic. Nature. 1988;331:341–343.

[2] Kolber ZS, Barber KH, Coale SE, Fitzwater SE, Greene RM, Johnson KS, et al Iron limitation of phytoplankton photosynthesis in the Equatorial Pacific Ocean. Nature. 1994;371:145–149.

[3] Behrenfeld M, Westberry T, Boss E, O’Malley R, Siegel D, Wiggert J, et al Satellite-detected fluorescence reveals global physiology of ocean phytoplankton. Biogeosciences. 2009;6:779–794.

[4] Behrenfeld MJ, Milligan AJ. Photophysiological expressions of iron stress in phytoplankton. Ann Rev Mar Sci. 2013;5:217–246. 10.1146/annurev-marine-121211-172356 22881354

[5] Boyd PW, Jickells T, Law CS, Blain S, Boyle EA, Buesseler KO, et al Mesoscale iron enrichment experiments 1993–2005: synthesis and future directions. Science. 2007;315:612–617. 10.1126/science.1131669 17272712

[6] Sunda WG, Huntsman SA. Interrelated influence of iron, light and cell size on marine phytoplankton growth. Nature. 1997;390:389–392.

[7] Strzepek RF, Harrison PJ. Photosynthetic architecture differs in coastal and oceanic diatoms. Nature. 2004;431:689–692. 10.1038/nature02954 15470428

[8] Petrou K, Trimborn S, Rost B, Ralph PJ, Hassler CS. The impact of iron limitation on the physiology of the Antarctic diatom *Chaetoceros simplex*. Marine biology. 2014;161:925–937. 10.1007/s00227-014-2392-z 24719494 PMC3969518

[9] La Roche J, Boyd PW., McKay RML, Geider RJ. Flavodoxin as an in situ marker for iron stress in phytoplankton. Nature. 1996;382:802–805.

[10] Peers G, Price NM. A role for manganese in superoxide dismutases and the growth of iron-deficient diatoms. Limnol Oceanogr. 2004;49:1774–1783.

[11] Peers G, Price NM. Copper-containing plastocyanin used for electron transport by an oceanic diatom. Nature. 2006;441:341–344. 10.1038/nature04630 16572122

[12] Marchetti A, Cassar N. Diatom elemental and morphological changes in response to iron limitation: a brief review with potential paleoceanographic applications. Geobiology. 2009;7:419–431. 10.1111/j.1472-4669.2009.00207.x 19659798

[13] Groussman RD, Parker MS, Armbrust EV. Diversity and Evolutionary History of Iron Metabolism Genes in Diatoms. PLoS ONE. 2015;10:e0129081 10.1371/journal.pone.0129081 26052941 PMC4460010

[14] Kustka AB, Allen AE, Morel FMM. Sequence analysis and transcriptional regulation of iron acquisition genes in two marine diatoms. J Phycol. 2007;43:715–729.

[15] Shaked Y, Kustka AB, Morel FMM. A general kinetic model for iron acquisition by eukaryotic phytoplankton. Limnol Oceanogr. 2005;50:872.

[16] Shaked Y, Lis H. Disassembling iron availability to phytoplankton. Front Microbiol. 2012;3:123 10.3389/fmicb.2012.00123 22529839 PMC3328120

[17] Morrissey J, Sutak R, Paz-Yepes J, Tanaka A, Moustafa A, Veluchamy A, et al A novel protein, ubiquitous in marine phytoplankton, concentrates iron at the cell surface and facilitates uptake. Curr Biol. 2015;25:364–371. 10.1016/j.cub.2014.12.004 25557662

[18] Botebol H, Lesuisse E, Šuták R, Six C, Lozano JC, Schatt P, et al Central role for ferritin in the day/night regulation of iron homeostasis in marine phytoplankton. Proc Natl Acad Sci U S A. 2015;112:14652–14657. 10.1073/pnas.1506074112 26553998 PMC4664360

[19] Allen AE, Laroche J, Maheswari U, Lommer M, Schauer N, Lopez PJ, et al Whole-cell response of the pennate diatom *Phaeodactylum tricornutum* to iron starvation. Proc Natl Acad Sci U S A. 2008;105:10438–10443. 10.1073/pnas.0711370105 18653757 PMC2492447

[20] Lommer M, Specht M, Roy AS, Kraemer L, Andreson R, Gutowska MA, et al Genome and low-iron response of an oceanic diatom adapted to chronic iron limitation. Genome Biol. 2012;13:R66 10.1186/gb-2012-13-7-r66 22835381 PMC3491386

[21] Thamatrakoln K, Korenovska O, Niheu AK, Bidle KD. Whole-genome expression analysis reveals a role for death-related genes in stress acclimation of the diatom *Thalassiosira pseudonana*. Environ Microbiol. 2012;14:67–81. 10.1111/j.1462-2920.2011.02468.x 21453404

[22] Bidle KD, Bender SJ. Iron starvation and culture age activate metacaspases and programmed cell death in the marine diatom *Thalassiosira pseudonana*. Eukaryot Cell. 2008;7:223–236. 10.1128/EC.00296-07 18039944 PMC2238155

[23] Nunn BL, Faux JF, Hippmann AA, Maldonado MT, Harvey HR, Goodlett DR, et al Diatom proteomics reveals unique acclimation strategies to mitigate Fe limitation. PLoS ONE. 2013;8:e75653 10.1371/journal.pone.0075653 24146769 PMC3797725

[24] Blankenship RE. Origin and evolution of photosynthesis In: Blankenship RE, ed. Molecular mechanisms of photosynthesis Oxford: Blackwell Science; 2002 pp. 220–257.

[25] Dlouhy AC, Outten CE. The iron metallome in eukaryotic organisms. Met Ions Life Sci 2013;12:241–278. 10.1007/978-94-007-5561-1_8 23595675 PMC3924584

[26] Raven JA. Predictions of Mn and Fe use efficiencies of phototrophic growth as a function of light availability for growth and of C assimilation pathway. New Phytol. 1990;116:1–18.

[27] Zinser ER, Lindell D, Johnson ZI, Futschik ME, Steglich C, Coleman ML, et al Choreography of the transcriptome, photophysiology, and cell cycle of a minimal photoautotroph, *Prochlorococcus*. PLoS ONE. 2009;4:e5135 10.1371/journal.pone.0005135 19352512 PMC2663038

[28] Ashworth J, Coesel S, Lee A, Armbrust EV, Orellana MV, Baliga NS. Genome-wide diel growth state transitions in the diatom *Thalassiosira pseudonana*. Proc Natl Acad Sci U S A. 2013;110:7518–7523. 10.1073/pnas.1300962110 23596211 PMC3645528

[29] Poliner E, Panchy N, Newton L, Wu G, Lapinsky A, Bullard B, et al Transcriptional coordination of physiological responses in *Nannochloropsis oceanica* CCMP1779 under light/dark cycles. Plant J. 2015;83:1097–1113. 10.1111/tpj.12944 26216534

[30] Zones JM, Blaby IK, Merchant SS, Umen JG. High-Resolution Profiling of a Synchronized Diurnal Transcriptome from *Chlamydomonas reinhardtii* Reveals Continuous Cell and Metabolic Differentiation. Plant Cell. 2015;27:2743–2769. 10.1105/tpc.15.00498 26432862 PMC4682324

[31] Chisholm SW, Costello JC. Influence of environmental factors and population composition on the timing of cell division in *Thalassiosira fluviatilis* (Bacillariophyceae) grown on light/dark cycles. J Phycol. 1980;16:375–383.

[32] Chisholm SW. Temporal patterns of cell-division in unicellular algae. Can B Fish Aquat Sci. 1981;210:150–181.

[33] Vaulot D. The cell cycle of phytoplankton: coupling cell growth to population growth In: Joint I, ed. Molecular ecology of aquatic microbes. Verlag Berlin Heidelberg: Springer; 1995 pp. 303–322.

[34] Ragni M, d Alcala MR. Circadian variability in the photobiology of *Phaeodactylum tricornutum*: pigment content. J Plankton Res. 2007;29:141–156.

[35] Bruyant F, Babin M, Genty B, Prasil O, Behrenfeld MJ, Claustre H, et al Diel variations in the photosynthetic parameters of *Prochlorococcus* strain PCC 9511: combined effects of light and cell cycle. Limnol Oceangr. 2005;50:850–863.

[36] Huysman MJ, Vyverman W, De Veylder L. Molecular regulation of the diatom cell cycle. J Exp Bot. 2014;65:2573–2584. 10.1093/jxb/ert387 24277280

[37] Schrader PS, Milligan AJ, Behrenfeld MJ. Surplus photosynthetic antennae complexes underlie diagnostics of iron limitation in a cyanobacterium. PLoS ONE. 2011;6:e18753 10.1371/journal.pone.0018753 21533084 PMC3080375

[38] Behrenfeld MJ, Worthington K, Sherrell RM, Chavez FP, Strutton P, McPhaden M, et al Controls on tropical Pacific Ocean productivity revealed through nutrient stress diagnostics. Nature. 2006;442:1025–1028. 10.1038/nature05083 16943835

[39] Greene RM, Geider RJ, Falkowski PG. Effect of iron limitation on photosynthesis in a marine diatom. Limnol Oceangr. 1991;36:1772–1782.

[40] Grouneva I, Jakob T, Wilhelm C, Goss R. The regulation of xanthophyll cycle activity and of non-photochemical fluorescence quenching by two alternative electron flows in the diatoms *Phaeodactylum tricornutum* and *Cyclotella meneghiniana*. BBA-Bioenergetics. 2009;1787:929–938. 10.1016/j.bbabio.2009.02.004 19232316

[41] Eisenstadt D, Ohad I, Keren N, Kaplan A. Changes in the photosynthetic reaction centre II in the diatom *Phaeodactylum tricornutum* result in non-photochemical fluorescence quenching. Environ Microbiol. 2008;10:1997–2007. 10.1111/j.1462-2920.2008.01616.x 18397307

[42] Cruz S, Goss R, Wilhelm C, Leegood R, Horton P, Jakob T. Impact of chlororespiration on non-photochemical quenching of chlorophyll fluorescence and on the regulation of the diadinoxanthin cycle in the diatom *Thalassiosira pseudonana*. J Exp Bot. 2011;62:509–519. 10.1093/jxb/erq284 20876335 PMC3003802

[43] Lavaud J, van Gorkom HJ, Etienne AL. Photosystem II electron transfer cycle and chlororespiration in planktonic diatoms. Photosyn Res. 2002;74:51–59. 10.1023/A:1020890625141 16228544

[44] Langfelder P, Mischel PS, Horvath S. When is hub gene selection better than standard meta-analysis? PLoS ONE. 2013;8:e61505 10.1371/journal.pone.0061505 23613865 PMC3629234

[45] Langfelder P, Horvath S. WGCNA: an R package for weighted correlation network analysis. BMC Bioinformatics. 2008;9:559 10.1186/1471-2105-9-559 19114008 PMC2631488

[46] Oldham MC, Konopka G, Iwamoto K, Langfelder P, Kato T, Horvath S, et al Functional organization of the transcriptome in human brain. Nat Neurosci. 2008;11:1271–1282. 10.1038/nn.2207 18849986 PMC2756411

[47] Saito MA, Bertrand EM, Dutkiewicz S, Bulygin VV, Moran DM, Monteiro FM, Follows MJ, Valois FW, Waterbury JB. Iron conservation by reduction of metalloenzyme inventories in the marine diazotroph *Crocosphaera watsonii*. Proc Natl Acad Sci U S A. 2010;108:2184–2189. 10.1073/pnas.1006943108 PMC3038740 21248230

[48] Marchetti A, Parker MS, Moccia LP, Lin EO, Arrieta AL, Ribalet F, Murphy MEP, Maldonado MT, Ambrust EV. Ferritin is used for iron storage in bloom-forming marine pennate diatoms. Nature. 2009;457:467–470. 10.1038/nature07539 19037243

[49] Roualt TA. An ancient gauge for iron. Science. 2009;326:676–677. 19900922 10.1126/science.1181938 PMC2932448

[50] Kobayashi T, Nishizawa NK. Iron sensors and signals in response to iron deficiency. Plant Sci. 2014;224:36–43. 10.1016/j.plantsci.2014.04.002 24908504

[51] Clemens S, Kim EJ, Neumann D, Schroeder JI. Tolerance to toxic metals by a gene family of phytochelatin synthases from plants and yeast. EMBO J. 1999;18:3325–3333. 10.1093/emboj/18.12.3325 10369673 PMC1171413

[52] Halliwell B, Gutteridge JM. Biologically relevant metal ion-dependent hydroxyl radical generation. An update. FEBS Lett. 1992;307:108–112. 1322323 10.1016/0014-5793(92)80911-y

[53] Shemi A, Ben-Dor S, Vardi A. Elucidating the composition and conservation of the autophagy pathway in photosynthetic eukaryotes. Autophagy. 2015;11:701–715. 10.1080/15548627.2015.1034407 25915714 PMC4502668

[54] Rayko E, Maumus F, Maheswari U, Jabbari K, Bowler C. Transcription factor families inferred from genome sequences of photosynthetic stramenopiles. New Phytol. 2010;188:52–66. 10.1111/j.1469-8137.2010.03371.x 20646219

[55] Brown MS, Ye J, Rawson RB, Goldstein JL. Regulated intramembrane proteolysis: a control mechanism conserved from bacteria to humans. Cell. 2000;4:391–398. 10.1016/s0092-8674(00)80675-3 10693756

[56] Guglielmi B, La Rochelle N, Tijan R. Gene-specific transcriptional mechanisms at the histone gene cluster revealed by single-cell imaging. Mol Cell. 2013;51:480–492. 10.1016/j.molcel.2013.08.009 23973376 PMC7659890

[57] Smith SR, Glé C, Abbriano RM, Traller JC, Davis A, Trentacoste E, Vernet M, Allen AE, Hildebrand M. Transcript level coordination of carbon pathways during silicon starvation-induced lipid accumulation in the diatom *Thalassiosira pseudonana*. New Phytol. 2016;210:890–904. 10.1111/nph.13843 26844818 PMC5067629

[58] Huysman MJJ, Martens C, Vyverman W, De Veylder L. Protein degradation during the diatom cell cycle: Annotation and transcriptional analysis of SCF and APC/C ubiquitin ligase genes in *Phaeodactylum tricornutum*. Mar Genomics. 2014;14:39–46. 10.1016/j.margen.2013.09.001 24055261

[59] Warner JR. The economics of ribosome biosynthesis in yeast. Trends Biochem Sci 1999;24:437–440. 10542411 10.1016/s0968-0004(99)01460-7

[60] Brauer MJ, Huttenhower C, Airoldi EM, Rosenstein R, Matese JC, Gresham D, et al Coordination of growth rate, cell cycle, stress response, and metabolic activity in yeast. Mol Biol Cell. 2008;19:352–367. 10.1091/mbc.E07-08-0779 17959824 PMC2174172

[61] Bowler C, Allen AE, Badger JH, Grimwood J, Jabbari K, Kuo A, Maheswari U, Martens C, et al The *Phaeodactylum* genome reveals the evolutionary history of diatom genomes. Nature. 2008;456:239–244. 10.1038/nature07410 18923393

[62] Huysman MJJ, Martens C, Vandepoele K, Gillard J, Rayko E, Heijde M, et al Genome-wide analysis of the diatom cell cycle unveils a novel type of cyclins involved in environmental signaling. Genome Biol. 2010;11:R17 10.1186/gb-2010-11-2-r17 20146805 PMC2872877

[63] Huysman MJJ, Tanaka A, Bowler C, Vyverman W, De Veylder L. Functional characterization of the diatom cyclin-dependent kinase A2 as a mitotic regulator reveals plant-like properties in a non-green lineage. BMC Plant Biol. 2015;15:86 10.1186/s12870-015-0469-6 25887918 PMC4392632

[64] Huysman MJJ, Fortunato AE, Matthijs M, Schellenberger Costa B, Vanderhaeghen R, Van den Daele H, Sachse M, Inzé D, Bowler C, Kroth PG, Wilhelm C, Falciatore A, Vyverman W, De Veylder L. AUREOCHROME1a-mediated induction of the diatom-specific cyclin dsCYC2 controls the onset of cell division in diatoms (*Phaeodactylum tricornutum*). 2013;25:215–228. 10.1105/tpc.112.106377 23292736 PMC3584536

[65] Noordally ZB, Millar AJ. Clocks in algae. Biochemistry. 2015;54:171–183. 10.1021/bi501089x 25379817

[66] Depauw FA, Rogato A, Ribera d’Alcalá M, Falciatore A. Exploring the molecular basis of responses to light in marine diatoms. J Exp Bot. 2012;63:1575–1591. 10.1093/jxb/ers005 22328904

[67] Hildebrand M. Diatoms, biomineralization processes, and genomics. Chem Rev. 2008;108:4855–4874. 10.1021/cr078253z 18937513

[68] Wilken S, Hoffman B, Hersch N, Kirchgessner N, Dieluweit S, Rubner W, et al Diatom frustules show increased mechanical strength and altered valve morphology under iron limitation. Limnol Oceanogr. 2011;56:1399–1410.

[69] Hutchins DA, Bruland KW. Iron-limited diatom growth and Si:N uptake ratios in a coastal upwelling regime. Nature. 1998;393:561–564.

[70] Assmy P, Smetacek V, Montresor M, Klaas C, Henjes J, Strass VH, et al Thick-shelled, grazer-protected diatoms decouple ocean carbon and silicon cycles in the iron-limited Antarctic Circumpolar Current. Proc Natl Acad Sci U S A. 2013;110:20633–20638. 10.1073/pnas.1309345110 24248337 PMC3870680

[71] Dugdale RC, Wilkerson FP. Silicate regulation of new production in the equatorial Pacific upwelling. Nature. 1998;391:3.9422496

[72] Brzezinski MA, Krause JW, Bundy RM, Barbeau KA, Franks P, Goericke R, Landry MR, Stukel MR. Enhanced silica ballasting from iron stress sustains carbon export in a frontal zone within the California Current. J Geophys Res. 2015;120:4654–4669.

[73] Barber J, Andersson B. Too much of a good thing: light can be bad for photosynthesis. Trends Biochem Sci. 1992;17:61–66. 1566330 10.1016/0968-0004(92)90503-2

[74] Koziol AG, Borza T, Ishida K, Keeling P, Lee RW, Durnford DG. Tracing the evolution of the light-harvesting antennae in chlorophyll a/b-containing organisms. Plant Physiol. 2007;143:1802–1816. 10.1104/pp.106.092536 17307901 PMC1851817

[75] Nymark M, Valle KC, Hancke K, Winge P, Andresen K, Johnsen G, et al Molecular and photosynthetic responses to prolonged darkness and subsequent acclimation to re-illumination in the diatom *Phaeodactylum tricornutum*. PLoS ONE. 2013; 8:e58722 10.1371/journal.pone.0058722 23520530 PMC3592843

[76] Coesel S, Mangogna M, Ishikawa T, Heijde M, Rogato A, Finazzi G, Todo T, Bowler C, Falciatore A. Diatom PtCPF1 is a new cryptochrome/photolyase family member with DNA repair and transcription regulation activity. EMBO Rep. 2009;10:655–661. 10.1038/embor.2009.59 19424294 PMC2711838

[77] Gillard J, Devos V, Huysman MJ, De Veylder L, D’Hondt S, Martens C, et al Physiological and transcriptomic evidence for a close coupling between chloroplast ontogeny and cell cycle progression in the pennate diatom *Seminavis robusta*. Plant Physiol. 2008;148:1394–1411. 10.1104/pp.108.122176 18820084 PMC2577256

[78] Peers G, Truong TB, Ostendorf E, Busch A, Elrad D, Grossman AR, et al An ancient light-harvesting protein is critical for the regulation of algal photosynthesis. Nature. 2009;462:518–521. 10.1038/nature08587 19940928

[79] Bailleul B, Rogato A, de Martino A, Coesel S, Cardol P, Bowler C, et al An atypical member of the light-harvesting complex stress-related protein family modulates diatom responses to light. Proc Natl Acad Sci U S A. 2010;107:18214–18219. 10.1073/pnas.1007703107 20921421 PMC2964204

[80] Zhu SH, Green BR. Photoprotection in the diatom *Thalassiosira pseudonana*: role of LI818-like proteins in response to high light stress. Biochim Biophys Acta. 2010;1797:1449–1457. 10.1016/j.bbabio.2010.04.003 20388491

[81] Davis SJ, Bhoo SH, Durski AM, Walker JM, Vierstra RD. The heme-oxygenase family required for phytochrome chromophore biosynthesis is necessary for proper photomorphogenesis in higher plants. Plant Physiol. 2001;126:656–669. 11402195 10.1104/pp.126.2.656 PMC111157

[82] Tasaka Y, Gombos Z, Nishiyama Y, Mohanty P, Ohba T, Ohki K, et al Targeted mutagenesis of acyl-lipid desaturases in Synechocystis: evidence for the important roles of polyunsaturated membrane lipids in growth, respiration and photosynthesis. EMBO J 1996;15:6416–6425. 8978669 PMC452467

[83] Gombos Z, Kanervo E, Tsvetkova N, Sakamoto T, Aro EM, Murata N. Genetic Enhancement of the Ability to Tolerate Photoinhibition by Introduction of Unsaturated Bonds into Membrane Glycerolipids. Plant Physiol. 1997;115:551–559. 12223823 10.1104/pp.115.2.551 PMC158514

[84] Allakhverdiev SI, Kinoshita M, Inaba M, Suzuki I, Murata N. Unsaturated fatty acids in membrane lipids protect the photosynthetic machinery against salt-induced damage in *Synechococcus*. Plant Physiol. 2001;125:1842–1853. 11299364 10.1104/pp.125.4.1842 PMC88840

[85] Chauton MS, Winge P, Brembu T, Vadstein O, Bones AM. Gene regulation of carbon fixation, storage, and utilization in the diatom *Phaeodactylum tricornutum* acclimated to light/dark cycles. Plant Physiol. 2013;161:1034–1048. 10.1104/pp.112.206177 23209127 PMC3561001

[86] Moog D, Rensing SA, Archibald JM, Maier UG, Ullrich KK. Localization and evolution of putative triose phosphate translocators in the diatom *Phaeodactylum tricornutum*. Genome Biol Evol. 2015;7:2955–2969. 10.1093/gbe/evv190 26454011 PMC5635587

[87] Granum E, Kirkvold S, Myklestad SM. Cellular and extracellular production of carbohydrates and amino acids by the marine diatom *Skeletonema costatum*: diel variations and effects of N depletion. MEPS. 2002;242:83–94.

[88] Plaxton WC. The organization and regulation of plant glycolysis. Annu Rev Plant Biol. 1996;47:185–214. 10.1146/annurev.arplant.47.1.185 15012287

[89] Ginger ML, McFadden GI, Michels PAM. Rewiring and regulation of cross-compartmentalized metabolism in protists. Phil Trans R Soc B. 2010;365:831–845. 10.1098/rstb.2009.0259 20124348 PMC2817232

[90] Kroth PG, Chiovitti A, Gruber A, Martin-Jezequel V, Mock T, Parker MS, et al A model for carbohydrate metabolism in the diatom *Phaeodactylum tricornutum* deduced from comparative whole genome analysis. PLoS ONE. 2008;3:e1426 10.1371/journal.pone.0001426 18183306 PMC2173943

[91] Smith SR, Abbriano RM, Hildebrand M. Comparative analysis of diatom genomes reveals substantial differences in the organization of carbon partitioning pathways. Algal Res. 2012;1:2–16.

[92] Fabris M, Matthijs M, Rombauts S, Vyverman W, Goossens A, Baart GJE. The metabolic blueprint of *Phaeodactylum tricornutum* reveals a eukaryotic Entner-Doudoroff glycolytic pathway. Plant J. 2012;70:1004–1014. 10.1111/j.1365-313X.2012.04941.x 22332784

[93] Kim J, Fabris M, Baart G, Kim MK, Goossens A, Vyverman W, et al Flux balance analysis of primary metabolism in the diatom *Phaedactylum tricornutum*. Plant J. 2015;85:161–176. 10.1111/tpj.13081 26590126

[94] Bailleul B, Berne N, Murik O, Petroutsos D, Prihoda J, Tanaka A, et al Energetic coupling between plastids and mitochondria drives CO_2_ assimilation in diatoms. Nature. 2015;524:366–369. 10.1038/nature14599 26168400

[95] Allen AE, Moustafa A, Montsant A, Eckert A, Kroth PG, Bowler C. Evolution and functional diversification of fructose bisphosphate aldolase genes in photosynthetic marine diatoms. Mol Biol Evol. 2012;29:367–379. 10.1093/molbev/msr223 21903677 PMC3245544

[96] Halsey KH, O’Malley RTO, Graff JR, Milligan AJ, Behrenfeld MJ. A common partitioning strategy for photosynthetic products in evolutionarily distinct phytoplankton species. New Phytol. 2013;4:1030–1038. 10.1111/nph.12209 23452244

[97] Green MA, Fry SC. Vitamin C degradation in plant cells via enzymatic hydrolysis of 4-O-oxalyl-L-threonate. Nature. 2005;433:83–87. 10.1038/nature03172 15608627

[98] Englard S, Seifter S. The biochemical functions of ascorbic acid. Annu Rev Nutr. 1986;6:365–406. 10.1146/annurev.nu.06.070186.002053 3015170

[99] Simpson GLW, Ortwert BJ. The non-oxidative degradation of ascorbic acid at physiological conditions. Biochim Biophys Acta. 2000;1501:12–24. 10727845 10.1016/s0925-4439(00)00009-0

[100] Lane DJ, Chikhani S, Richardson V, Richardson DR. Transferrin iron uptake is stimulated by ascorbate via an intracellular reductive mechanism. Biochim Biophys Acta. 2013;1833:1527–1541. 10.1016/j.bbamcr.2013.02.010 23481043

[101] Lane DJ, Lawen A. Non-transferrin iron reduction and uptake are regulated by transmembrane ascorbate cycling in K562 cells. J Biol Chem. 2008;283:12701–12708. 10.1074/jbc.M800713200 18347019

[102] Guerinot ML. Microbial iron transport. Annu Rev Microbiol. 1994;48:743–772. 10.1146/annurev.mi.48.100194.003523 7826025

[103] Nunn BL, Faux JF, Hippmann AA, Maldonado MT, Harvey HR, Goodlett DR, et al Diatom proteomics reveals unique acclimation strategies to mitigate Fe limitation. PLoS ONE. 2013;8:e75653 10.1371/journal.pone.0075653 24146769 PMC3797725

[104] Ge F, Huang W, Chen Z, Zhang C, Xiong Q, Bowler C, et al Methycrotonyl-CoA carboxylase regulates triacylglycerol accumulation in the model diatom *Phaedactylum tricornutum*. Plant Cell. 2014;26:1681–1697. 10.1105/tpc.114.124982 24769481 PMC4036579

[105] Shtaida N, Khozin-Goldberg I, Boussiba S. The role of pyruvate hub enzymes in supplying carbon precursors for fatty acid synthesis in photosynthetic microalgae. Photosynth Res. 2015;3:407–422. 10.1007/s11120-015-0136-7 25846135

[106] Roberts K, Granum E, Leegood RC, Raven JA. C_3_ and C_4_ pathways of photosynthetic carbon assimilation in marine diatoms are under genetic, not environmental, control. Plant Physiol. 2007;145:230–235. 10.1104/pp.107.102616 17644625 PMC1976569

[107] Valenzuela J, Mazurie A, Carlson RP, Gerlach R, Cooksey KE, Peyton BM, et al Potential role of multiple carbon fixation pathways during lipid accumulation in *Phaeodactylum tricornutum*. Biotechnol Biofuels. 2012;5:40 10.1186/1754-6834-5-40 22672912 PMC3457861

[108] Davis A, Abbriano R, Smith SR, Hildebrand M. Clarification of photorespiratory processes and the role of malic enzyme in diatoms. Protist. 2016; in press. 10.1016/j.protis.2016.10.005 28104538

[109] Pate JS, Layzell DB. Energetics and biological costs of nitrogen assimilation In: Miflin BJ, Lea P, eds. The Biochemistry of Plants Vol 16. Intermediary Nitrogen Metabolism. San Diego: Academic Press Inc.; 1990 pp. 1–42.

[110] Brown KL, Twing KI, Robertson DL. Unraveling the regulation of nitrogen assimilation in the marine diatom *Thalassiosira pseudonana* (Bacillariophyceae): diurnal variations in transcript levels for five genes involved in nitrogen assimilation. J Phycol. 2009;45:413–426. 10.1111/j.1529-8817.2009.00648.x 27033820

[111] Vardi A, Formiggini F, Casotti R, De Martino A, Ribalet F, Miralto A, et al A stress surveillance system based on calcium and nitric oxide in marine diatoms. PLoS Biol. 2006;4:e60 10.1371/journal.pbio.0040060 16475869 PMC1370914

[112] Vardi A, Bidle KD, Kwityn C, Hirsh DJ, Thompson SM, Callow JA, et al A diatom gene regulating nitric-oxide signaling and susceptibility to diatom-derived aldehydes. Curr Biol. 2008;18:895–899. 10.1016/j.cub.2008.05.037 18538570

[113] Crawford NM, Guo FQ. New insights into nitric oxide metabolism and regulatory function. Trends Plant Sci. 2005;10:195–200. 10.1016/j.tplants.2005.02.008 15817421

[114] Berges JA, Harrison PJ. Relationships between nitrate reductase activity and rates of growth and nitrate incorporation under steady-state light or nitrate limitation in the marine diatom *Thalassiosira pseudonana* (Bacillariophyceae). J Phycol. 2008;31:85–95.

[115] Needoba JA, Harrison PJ. Influence of low light and a light:dark cycle on NO3- uptake, intracellular NO3’, and nitrogen isotope fractionation by marine phytoplankton. J Phycol. 2004;40:505–516.

[116] Bender SJ, Durkin CA, Berthiaume CT, Morales RL, Armbrust EV. Transcriptional responses of three model diatoms to nitrate limitation of growth. Front Mar Sci. 2014;1:3.

[117] Allen AE. Beyond sequence homology: redundant ammonium transporters in a marine diatom are not functionally equivalent. J Phycol. 2005;41:4–6.

[118] Allen AE, Dupont CL, Oborník M, Horák A, Nunes-Nesi A, McCrow JP, et al Evolution and metabolic significance of the urea cycle in photosynthetic diatoms. Nature. 2011;473:203–207. 10.1038/nature10074 21562560

[119] Bender SJ, Parker MS, Armbrust EV. Coupled effects of light and nitrogen source on the urea cycle and nitrogen metabolism over a diel cycle in the marine diatom *Thalassiosira pseudonana*. Protist. 2012;163:232–251. 10.1016/j.protis.2011.07.008 21873112

[120] Levering J, Broddrick J, Dupont CL, Peers G, Beeri K, Mayers J. Genome-scale model reveals metabolic basis of biomass partitioning in a model diatom. PLoS ONE. 2016;11:e0155038 10.1371/journal.pone.0155038 27152931 PMC4859558

[121] Caldovic L, Tuchman M. N-acetylglutamate and its changing role through evolution. Biochem J. 2003;372:279–290. 10.1042/BJ20030002 12633501 PMC1223426

[122] Sunda WG, Price NM, Morel FMM. Trace metal ion buffers and their use in culture studies In: Anderson RA, editor. Algal Culturing Techniques. Oxford: Elsevier Academic Press; 2005 p. 35–63.

[123] Samuelsson G, Öquist G. A method for studying photosynthetic capacities of unicellular algae based on in vivo chlorophyll fluorescence. Physiol Plant. 1977;40:315–319.

[124] Tavazoie S, Hughes JD, Campbell MJ, Cho RJ, Church GM. Systematic determination of genetic network architecture. Nat Genet. 1999;281–285. 10.1038/10343 10391217

[125] Chatfield M, Mander A. The Skillings-Mack test (Friedman test when there are missing data). Stata J. 2009;9:299–305. 19829764 PMC2761045

[126] Barrera L, Benner C, Tao YC, Winzeler E, Zhou Y. Leveraging two-way probe-level block design for identifying differential gene expression with high-density oligonucleotide arrays. BMC Bioinformatics. 2004;5:42 10.1186/1471-2105-5-42 15099405 PMC411067

[127] Wang H, He X. An enhanced quantile approach for assessing differential gene expressions. Biometrics. 2008;64:449–457. 10.1111/j.1541-0420.2007.00903.x 18325069

[128] McCarthy DJ, Chen Y, Smyth GK. Differential expression analysis of multifactor RNA-Seq experiments with respect to biological variation. Nucleic Acids Res. 2012;40:4288–4297. 10.1093/nar/gks042 22287627 PMC3378882

[129] Skillings JH, Mack GA: On the use of a Friedman-type statistic in balanced and unbalanced block designs. Technometrics 1981, 23:171–177.

[130] Saeed AI, Bhagabati NK, Braisted JC, Liang W, Sharov V, Howe EA, et al TM4 microarray software suite. Meth Enzymol 2006, 411:134–193.—McCarthy? 10.1016/S0076-6879(06)11009-5 16939790

[131] Maere S, Heymans K, Kuiper M: BiNGO: a Cytoscape plugin to assess overrepresentation of gene ontology categories in biological networks. Bioinformatics 2005, 21:3448–3449. 10.1093/bioinformatics/bti551 15972284

[132] Gruber A, Weber T, Bártulos CR, Vugrinec S, Kroth PG. Intracellular distribution of the reductive and oxidative pentose phosphate pathways in two diatoms. J Basic Microbiol. 2009;49:58–72. 10.1002/jobm.200800339 19206144

[133] Bendtsen JD, Nielse H, von Heijne G, Brunak S. Improved prediction of signal peptides: SignalP 3.0. J Mol Biol 2004:340:783–795. 10.1016/j.jmb.2004.05.028 15223320

[134] Petersen TN, Brunak S, von Heijne G, Nielsen H. SignalP 4.0: discriminating signal peptides from transmembrane proteins. Nat Meth. 2011;8:785–786. 10.1038/nmeth.1701 21959131

[135] Gruber A, Rocap G, Kroth PG, Armbrust EV, Mock T. Plastid proteome prediction for diatoms and other algae with secondary plastids of the red lineage. Plant J. 2015;81:519–528. 10.1111/tpj.12734 25438865 PMC4329603

[136] Claros MG, Vincens P. Computational method to predict mitochondrially imported proteins and their targeting sequences. Eur J Biochem. 1996;241:779–786. 8944766 10.1111/j.1432-1033.1996.00779.x

[137] Krogh A, Larsson B, von Heijne G, Sonnhammer ELL. Predicting transmembrane protein topology with a hidden markov model: application to complete genomes. J Mol Biol. 2001;205:567–580. 10.1006/jmbi.2000.4315 11152613

[138] Lisec J, Schauer N, Kopka J, Willmitzer L, Fernie AR. Gas chromatography mass spectrometry-based metabolite profiling in plants. Nat Protoc. 2006;1:387–396. 10.1038/nprot.2006.59 17406261

[139] Schauer N, Steinhauser D, Strelkov S, Schomburg D, Allison G, Moritz T, et al GC-MS libraries for the rapid identification of metabolites in complex biological samples. FEBS Lett. 2005;579:1332–1337. 10.1016/j.febslet.2005.01.029 15733837

