# Correction: Transcriptional Orchestration of the Global Cellular Response of a Model Pennate Diatom to Diel Light Cycling under Iron Limitation

**DOI:** 10.1371/journal.pgen.1006688

**Published:** 2017-03-29

**Authors:** Sarah R. Smith, Jeroen T. F. Gillard, Adam B. Kustka, John P. McCrow, Jonathan H. Badger, Hong Zheng, Ashley M. New, Chris L. Dupont, Toshihiro Obata, Alisdair R. Fernie, Andrew E. Allen

There are errors in S1 Dataset, S2 Dataset, S4 Dataset and S5 Dataset. Expression values reported in the S5 Dataset are not assigned to the correct gene identifiers. Please view the correct Datasets here, with updated cross references in the S1 Dataset, S2 Dataset and S4 Dataset.

## Supporting information

S1 Dataset Active transcriptome and assignment of genes to WGCNA modules and response types. (XLSX)

S2 Dataset List of highly low Fe responsive genes. (XLSX)

S4 Dataset Metabolic pathway annotations. (XLSX)

S5 Dataset Phatr3 and Phatr2 comparison. (XLSB)
