# Supplementary material for: Transcriptional Orchestration of the Global Cellular Response of a Model Pennate Diatom to Diel Light Cycling under Iron Limitation
Source: PLoS Genet. 2016 Dec 14;12(12):e1006490. doi: 10.1371/journal.pgen.1006490 (PMC5156380; doi:10.1371/journal.pgen.1006490)
Supplement: S15 Fig — (A) Table summarizing targeting predictions (see methods) for both NAGS1 and NAGS2 gene candidates. (B) Sequences used for gene targeting, with Phatr3 gene model ID and EST-based N-term extensions (NAGS1) indicated. (C) Domain architecture (from NCBI blastp) showing hits to PRK05279 (N-acetylglutamate synthase, validated). (PDF) [file pgen.1006490.s020.pdf]

A Targeting predictions

	Gene Model	Signal P	Cleavage	ASAFIND	Mitoprot	TMHMM	Verdict
NAGS1	Phatr3_J10902.t1 (+108aa)	v3.0: N (0.025)*<br>v4.1 N (D = 0.254)	--<br>--	--	0.9489	Yes<br>44-63	TM domain /plastid
	Phatr3_J10902.t1 (+72aa)	v3.0: Y (0.814)<br>v4.1: N (D = 0.384)	TLA-TL<br>--	Y – low conf.<br>--	0.2666	No	
	Phatr3_J10902.t1	v3.0: N (0.025)<br>v4.1: N (D = 0.098)	--<br>--	--<br>--	0.0029	No	
NAGS2	Phatr3_J44492.t1	v3.0: Y (0.977)<br>v4.1: Y (D = 0.705)	SYT-GI<br>AKS-YT	N<br>N	0.6859	No	Signal peptide

*Signal anchor probability = 0.916

B Sequences

Transmembrane Domain
EST extended model
PRK05279 Domain

NAGS1

>Phatr3_J10902.t1_ESTextended

```
MNTAIVVYRQQLRLLQLLTVSSRSPPLRSWRGLSSKMKTSTHKVFGLSTWVVATLATLPIHWITESFVPSALPLNQKSPSLRRPSLASFPVFPNPLPITTASSS
SPLHMAATDKEQQLDAAANTKHAATAPKGNEHSNVGHHSSLSNVGGKAPKDFHVDHEAGLRVDGRSSPQRHLSSSTTKESTLSPQNVTFEAQKPKGYSTFDASS
LLAVDPSCINIDTVPPSEEEFGPPTEEALKRLPFVSMFRGSANYIANHRNTLAVYHIPGGLLDQPGNNVFRDLMNDIALTWLLGMKIVLVVGCRHQIAKRVAQH
QSDSSLEENLQSRHGLRVTDEKTLRIVKEEAGYVRFEVERQLARSLRMQGGGMGNAPGVGSTGQGYYDGNVVSGNFYSAQPFGILDGVDFKYTGFVRRVEVEWI
RQKHNNRDICLMTTLGVSPSGEVFNVNSEALAASVAGALEASKVIYFTEQEMELRHTQHGKKIQSLRMSDANNLLEYSGVTIHKKGFVQLDDELHEMDKAALDM
LLKIGWSVQALEMGVKRAHIISPKHGALLQELYTRDGSGLLISGDLYDGIRRANVHDVAAIHDLISPLIQMGTLIDRPKSVLEKDVESYYVFTRDNHIVASGQL
KIFENGFAEIGCLVVNQGYRSRGRGDAMLGYLERLCLVNSCSSIFVLSTQTMEWFVERGFDEVGVDRLPPSRQATYNYKRNSKIYMKKIESDRDLDASELWWNR
```

NAGS2

>Phatr3_J44492.t1

```
MVMLSLIAFVISTWVAKSYTGIPKSASVRHPHTGPRATGSFTVNVRKSSIAYQFSASTESSDFSTWDGSAPLSVDDSYDELVTGALQISPLSVNDPALVEFTES
THFRKSVRNGKVLPAMGSSHYPFAMMLQGSAPYIAAHAGKTAVIHIPGDVLDGTPNADALLADISLSRLLGMKIVIVIGCRFDLDRCDMDFAENAHECHNALKI
TNAETLRYVEEEVGFLRTEMERKLNRCLRMTGGVSGTKDAPVPDGNVVSGNFYTAQQFGIIQGEDFEYTGFTPTGQVHAENIQRVLNNNDVVLLTTVGLSTLGE
LVNVNGYHLAASVAAALKAYKVIYMSNEGSVLQKKGENISIQELPLSFAKAVLDYHHVEVHKTGFATFERARQSLEPHAVELLLHLGWASWALDHGVKRAHVVN
PGDGALLEELFTSKNGANTCLYHDEENTKVEEEIGQDDWDSFFASAKAQGQKEIYIKKQQSSKRF
```

C Domain architecture

NAGS1

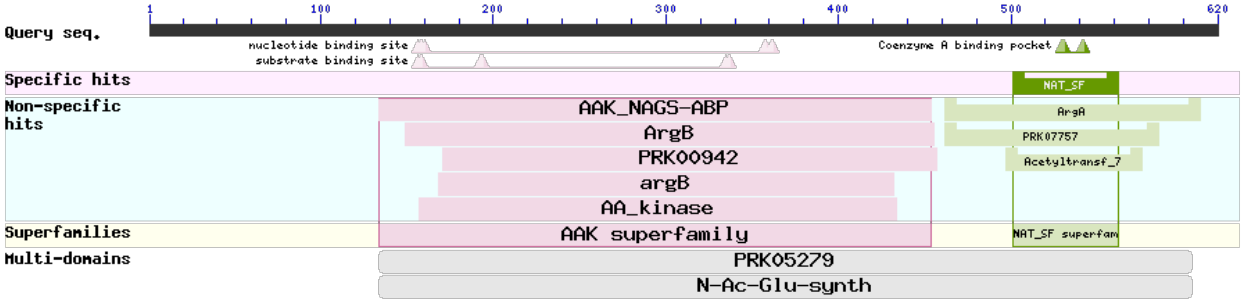

NAGS2

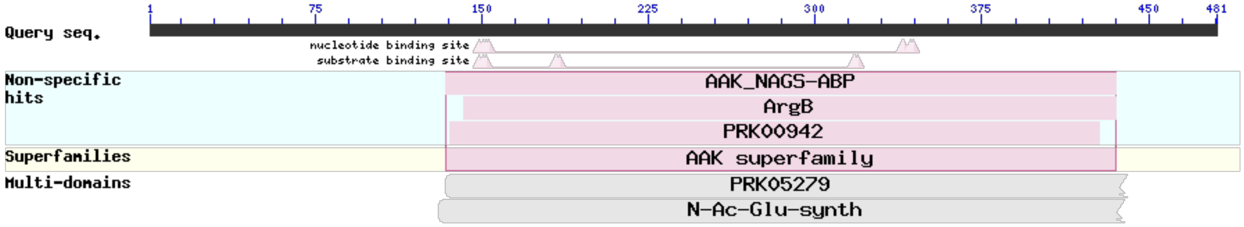

S7 Fig
