# Supplementary material for: Transcriptional Orchestration of the Global Cellular Response of a Model Pennate Diatom to Diel Light Cycling under Iron Limitation
Source: PLoS Genet. 2016 Dec 14;12(12):e1006490. doi: 10.1371/journal.pgen.1006490 (PMC5156380; doi:10.1371/journal.pgen.1006490)
Supplement: S1 Table — (PDF) [file pgen.1006490.s023.pdf]

S1 Table. Experimental Design. *P. tricornutum* was grown on a 12:12 light:dark regime. Lights were on at 9AM, and off at 9PM, therefore cultures were sampled 3 hours before (6AM/6PM) and 1 hour (10AM/10PM) after each light transition.

	[Fe]		Day 1				Day 2			
**Experiment**		**pM**	**10AM**	**2PM**	**6PM**	**10PM**	**2AM**	**6AM**	**10AM**	**2PM**
**L1**	15	27		X	X	X	X	X	X	X
**L2**	15	27	X	X	X	X	X	X	X	
**M1**	30	54		X	X	X	X	X	X	X
**M2**	30	54	X	X	X	X	X	X	X	
**H1**	300	540		X	X	X	X	X	X	X
**H2**	300	540	X	X	X	X	X	X	X	
**H3**	300	540	X	X	X	X	X	X	X	

**Sample (n) Totals**			**10AM**	**2PM**	**6PM**	**10PM**	**2AM**	**6AM**
**L Total**	15	27	*3*	*3*	*2*	*2*	*2*	*2*
**M Total**	30	54	*3*	*3*	*2*	*2*	*2*	*2*
**H Total**	300	540	*4*	*5*	*3*	*3*	*3*	*3*
Combined Total			***10***	***11***	***7***	***7***	***7***	***7***
